# Photo-crosslink analysis in nonribosomal peptide synthetases reveals aberrant gel migration of branched crosslink isomers and spatial proximity between non-neighboring domains†

**DOI:** 10.1039/d0sc01969k

**Published:** 2020-08-11

**Authors:** Eva Dehling, Jennifer Rüschenbaum, Julia Diecker, Wolfgang Dörner, Henning D. Mootz

**Affiliations:** Institute of Biochemistry, Department of Chemistry and Pharmacy, University of Muenster D-48149 Münster Germany Henning.Mootz@uni-muenster.de

## Abstract

Nonribosomal peptide synthetases (NRPSs) are large, multi-modular enzyme templates for the biosynthesis of important peptide natural products. Modules are composed of a set of semi-autonomous domains that facilitate the individual reaction steps. Only little is known about the existence and relevance of a higher-order architecture in these mega-enzymes, for which contacts between non-neighboring domains in three-dimensional space would be characteristic. Similarly poorly understood is the structure of communication-mediating (COM) domains that facilitate NRPS subunit docking at the boundaries between epimerization and condensation domains. We investigated a COM domain pair in a minimal two module NRPS using genetically encoded photo-crosslinking moieties in the N-terminal acceptor COM domain. Crosslinks into the C-terminal donor COM domain of the partner module resulted in protein products with the expected migration behavior on SDS-PAGE gels corresponding to the added molecular weight of the proteins. Additionally, an unexpected apparent high-molecular weight crosslink product was revealed by mass spectrometric analysis to represent a T-form isomer with branched connectivity of the two polypeptide chains. Synthesis of the linear L-form and branched T-form isomers by click chemistry confirmed this designation. Our data revealed a surprising spatial proximity between the acceptor COM domain and the functionally unrelated small subdomain of the preceding adenylation domain. These findings provide an insight into three-dimensional domain arrangements in NRPSs in solution and suggest the described photo-crosslinking approach as a promising tool for the systematic investigation of their higher-order architecture.

## Abstract 2

Photo-crosslink analysis reveals unexpected insights into the higher-order architecture of NRPS and the nature of crosslink isomers.

## Graphical abstract

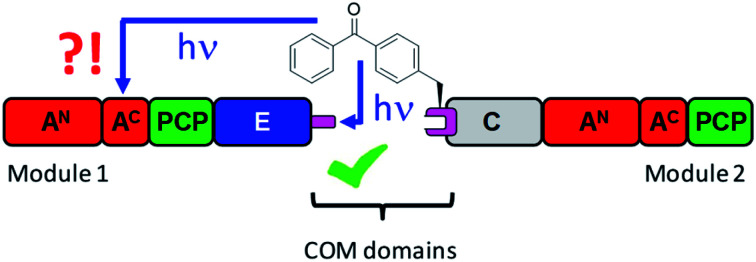

## Introduction

Nonribosomal peptide synthetases (NRPSs) are the protein templates for the biosynthesis of a huge variety of small bioactive peptide natural products, also referred to as nonribosomal peptides (NRPs).^1–6^ NRPs can act as antibiotics, siderophores, antifungal and antitumor drugs, for example, and include important compounds like vancomycin and cyclosporine. NRPSs are subdivided into modules, with typically each module being responsible for the incorporation of one amino acid into the peptide sequence. Modules contain specialized domains that activate the amino acid under consumption of ATP, bind them covalently as thioester at a prosthetic 4′-phosphopantetheine group (Ppant) of the peptidyl carrier protein (PCP), and catalyze peptide bond formation with the residue bound to the adjacent module. Once the linear product is assembled, it is cleaved off from the enzyme template by one of several mechanisms, including hydrolysis or reductive cleavage of the thioester as well as, most prominently, cyclization with the N terminus or a side chain of the peptide sequence. The essential adenylation (A), PCP, condensation (C), and thioesterase (TE) domains that are responsible for these reactions can be further supplemented with optional domains that carry out chemical modification of the building blocks at various steps during the assembly, for example epimerization and *N*-methylation catalyzed by E and M domains.

In a typical linear NRPS,^7^ the modules are arranged in a co-linear fashion with the amino acid sequence of the NRP product. While this arrangement suggests an ordered and largely repetitive spatial organization of modules and domains, it still remains unclear if and how multi-modular NRPSs templates are organized in a three-dimensional, higher-order superstructure.^8–12^ Crystal structures were obtained of several didomain truncation constructs^13–16^ and of some mono- and di-modules with multi-domain arrangements.^8,9,17–19^ These structural insights have confirmed significant conformational domain mobility between neighboring domains. In particular, the PCP domain as a carrier of covalently bound substrates and intermediates travels large distances to reach the various catalytic centers of the domains it has to interact with. In solution FRET studies have correlated these motions with catalysis.^20^ A recent cryo electron microscopy study suggested that the domain and module arrangements in multi-domain constructs might be very flexible, on top of the mobility of individual domains.^8^ Crystal structures of a dimodular fragment revealed contacts between a formylation domain of module 1 and a C domain of module 2, the first example of domain–domain contacts of domains that are not neighboring in the primary sequence.^9^ Such contacts might be indicative for a superstructure, however, due to crystal packing effects their occurrence as crystallization artefacts cannot be ruled out. For these reasons, new and complementary techniques to probe the structure of multimodular and entire NRPS templates in solution are needed.

**Fig. 1:**
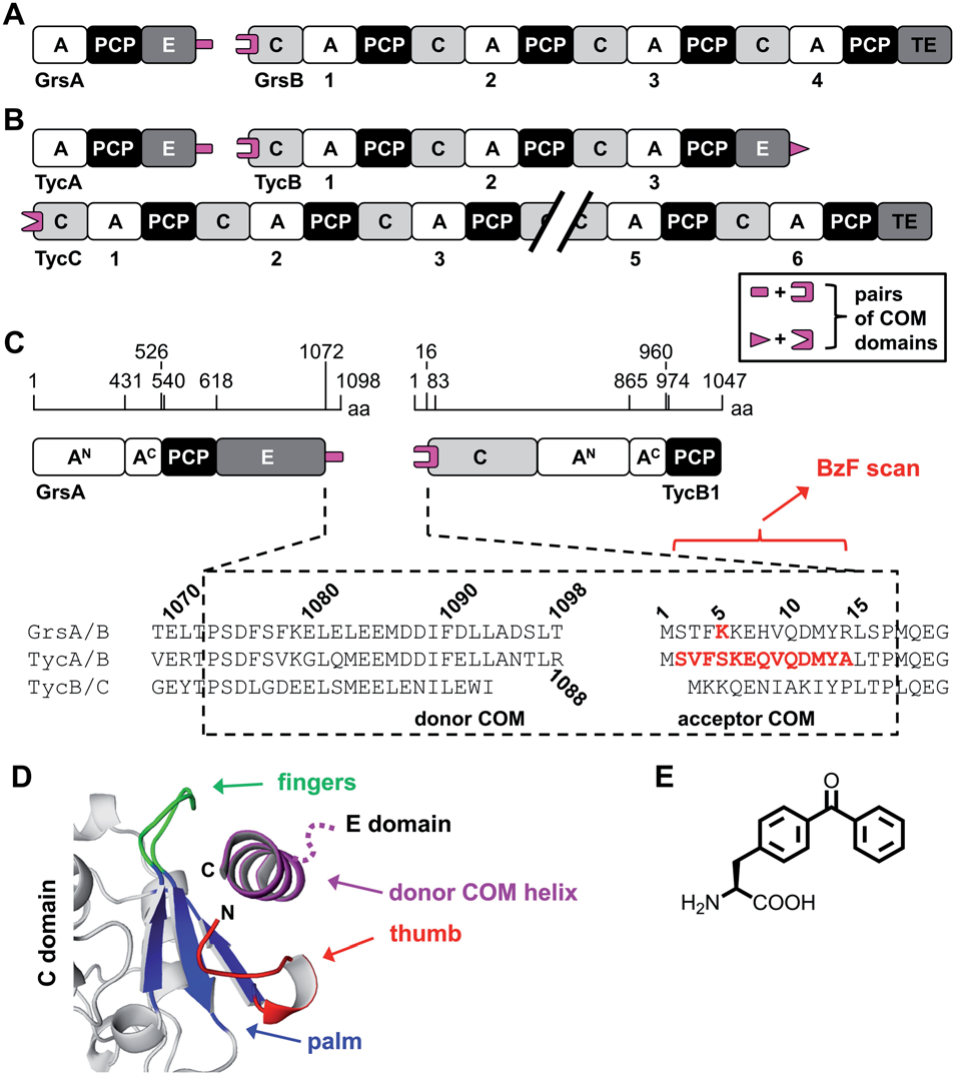
Representative NRPSs with COM domains. (A and B) Domain organization of the gramicidin S and tyrocidine synthetases, respectively. (C) Magnified illustration of the GrsA and TycB1 module with sequence alignment of the terminal COM domains. Numbering refers to the sequences of GrsA/TycA and GrsB1/TycB1 and indicated positions refer to domain boundaries according to initial studies.^21^ Amino acids in red were replaced with photo-crosslinking **BzF** (*p*-benzoyl-phenylalanine) in this study. (D) Model of the upside-down helix-hand model.^22^ Positions of the hand region are with respect to TycB1 numbering: aa2–8 (thumb), aa9–14 (first β sheet of palm), aa79–83 (second β sheet of palm), aa68–71 (third β sheet of palm), aa72–78 (fingers). The helix shown represents GrsA residues aa1081–1098. The illustration was created using pdb file 2VSQ, representing SrfA-C with an artificial tag helix.^17^ (E) Structure of **BzF**.

Another important aspect is the structural organization of NRPSs with more than one subunit, which is the predominant occurrence of bacterial NRPSs. For example, the five modules of the gramicidin S synthetase are distributed over two enzymes, the gramicidin S synthetase A (GrsA) with one module and the gramicidin S synthetase B (GrsB) with four modules (Fig. 1A). The interaction between subunits is facilitated by docking or communication-mediating (COM) domains.^21,23–28^ Multiple such interactions are required in NRPS templates with more than two subunits (Fig. 1B). Combinatorial exchange of docking and COM domains holds the potential to reprogram the biosynthetic pathways in order to obtain new peptide products.^21,23^ COM domains represent one specific class of docking domains that are found at NRPS subunit interfaces with an E domain at the upstream subunit and a C domain at the downstream subunit (Fig. 1A and B). This class was initially defined as C- and N-terminal tails of approx. 15–20 aa, referred to as donor and acceptor COM domains, respectively.^21,29^ Examples include the protein interaction interfaces between the GrsA and GrsB enzymes as well as between the pairs of TycA–TycB and TycB–TycC subunits in the tyrocidine NRPS (Fig. 1A–C). Initial biochemical and swapping studies suggested that these COM domains are the only mediators of the interaction and that the rather short peptide sequences possibly form helical structures.^21,29,30^

However, a different model was suggested by the crystal structure of a C domain with its N-terminal acceptor COM domain, as a part of the structure of the surfactin A synthetase C (SrfA-C) module. This structure showed by serendipity the binding of an unrelated tag sequence at the protein's C terminus to the acceptor COM domain in the crystal lattice.^17^ The tag sequence had reasonable sequence homology to the cognate donor COM domain, suggesting it mimicked the binding of the latter in the COM complex. The tag sequence adopted an α helix and formed contacts not only with the acceptor COM domain, but also with an extended surface on the C domain grasping around the helix like a hand, suggesting that additional sequences outside the COM regions might be important for the interactions. Based on this structure, a helix-hand model was proposed for the interaction (Fig. 1D).^17^ We could further support the general architecture of a helix-hand motif by a mutational and photo-crosslinking study of the donor COM domain of GrsA.^22^ However, based on spatial constraints obtained from mapped crosslinks, we predicted an upside-down orientation of the donor COM helix in the acceptor COM hand motif (Fig. 1D).^22^ Consistent with this revised model, a crystal structure of a C domain of the fungal TqaA NRPS later showed that the hand motif can bind an extended sequence as an α helix in such reverse orientation.^31^ Despite these studies, the actual structure of a COM domain complex remains elusive. Other swapping attempts showed mixed successes and thereby further underline that COM domains are not yet sufficiently understood to reliably reprogram NRPS templates.^32,33^

Genetically encoded photo-crosslinking amino acids have enabled the probing of protein–protein interaction interfaces in a position-dependent manner.^34^ The benzophenone moiety of the unnatural amino acid *p*-benzoyl-phenylalanine (**BzF**) is a widely used photo-crosslinker that can be repeatedly activated with light of approx. 350–365 nm.^35^ The formed diradical is short-lived and typically inserts into C–H bonds of side chains and the peptide backbone in a distance range of 3.1 Å,^36^ although larger labeling radii are possible due to rotations and flexibility at the **BzF** side chain and the surrounding environment.^35,37^ Methionine side chains have been observed as preferred crosslink partners.^38^

In this work, we have further investigated the architecture of a COM domain complex by using photo-crosslinking and mass spectrometry (MS) mapping of the crosslinks. We performed a positional scan with **BzF**^39^ in the acceptor COM domain of TycB1 in the dimodular GrsA–TycB1 system (Fig. 1C). We report the discovery of an unusual type of crosslink that produced a protein band with aberrantly slow migration behavior in SDS gel electrophoresis. By MS mapping and defined conjugate synthesis using bioorthogonal chemistry we show the importance of L-form and T-form crosslink isomers of the >250 kDa complex to explain the unusual migration behavior. Furthermore, our data suggests the spatial proximity of an unrelated catalytic adenylation domain to the COM interaction interface and thereby highlights the photo-crosslinking approach as a new method to study the higher-order architecture of the giant multi-domain NRPS.

## Results and discussion

### The GrsA–TycB1 system and its postulated COM domains

The first two modules GrsA/TycA and GrsB1/TycB1 from the gramicidin S and tyrocidine NRPSs are functionally interchangeable in forming a d-Phe-Pro dipeptide.^22,40^ The sequence alignment in Fig. 1C shows a high similarity of their COM domain sequences, whereas they differ from the COM domain pair found between TycB and TycC, which is also functionally orthogonal.^21^ The donor COM domains encompass residues 1072–1098 of GrsA (1098 aa) and residues 1062–1088 of TycA (1088 aa). The acceptor COM domain of GrsB1/TycB1 is defined from residue 2 to 16 (the starting methionine is removed), however, the helix-hand model proposes that residues up to approx. position 83 are involved in the non-continuous hand part (Fig. 1D).^17,22^ In this study we focused on the GrsA/TycB1 pair for reasons of consistency with these previous studies and because of long-standing problems in the recombinant production of GrsB1. Of note, we later performed control experiments with the homologous GrsA/GrsB1 and TycA/TycB1 pairs that confirmed the major findings on the GrsA/TycB1 pair (see below). The proteins were produced by recombinant production in *E. coli* and carried a C-terminal His_6_ tag to facilitate purification by Ni-NTA chromatography. GrsA additionally contained an N-terminal SBP tag (streptavidin binding peptide).

**Fig. 2:**
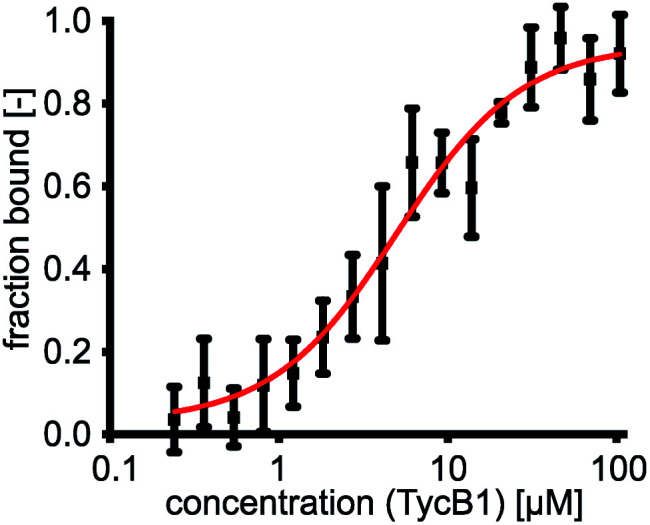
GrsA/TycB1 affinity determined by MST. GrsA was fluorescently labeled with NT-647-NHS ester (red dye). For MST (microscale thermophoresis) measurements 1 nM of labeled GrsA was incubated with a dilution series of TycB1(WT) as indicated. The experiment was performed in triplicate and error bars represent standard deviations.

We determined a dissociation constant *K*_d_ = (5.0 ± 0.9) μM for the interaction between GrsA and TycB1 using microscale thermophoresis (MST) (Fig. 2).

### Identification of different types of crosslink bands in the GrsA–TycB1(BzF) system

We employed incorporation of **BzF** and *p*-azidophenylalanine (**AzF**) by the genetic code expansion technology that relies on suppression of an amber stop codon through co-expression of an engineered pair of aminoacyl synthetase and tRNA in the presence of the unnatural amino acid.^39,41^ The idea of our photo-crosslinking approach is to identify residues involved in the interaction interface of the two proteins. A participating position would be revealed by either obtaining a crosslink with the photo-crosslinking amino acid incorporated at the respective position or by a position in the partner protein that is identified as the targeted residue of a crosslink. Mapping these positions on known or postulated structures allows the testing and refinement of structural models and possibly the proposal of new constraints of their architecture.

**Fig. 3:**
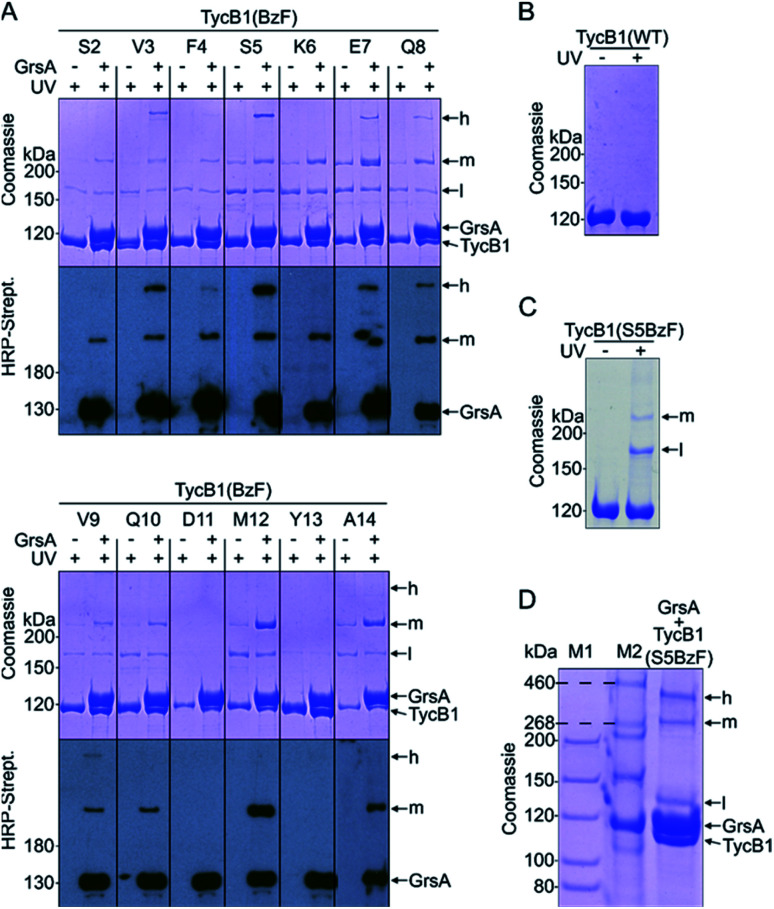
Photo-crosslinker scanning analysis. **BzF** was incorporated into residues 2–14 of the acceptor COM domain of TycB1. (A) Shown are UV-irradiated samples without or with the partner protein GrsA on coomassie-stained SDS-PAGE gels and immuno blots against the SBP-tag of GrsA. (B) Control experiment for UV-irradiation of wildtype TycB1. (C) Control experiment for UV-irradiation of TycB1(S5BzF). (D) Tris–acetate SDS-PAGE of a UV-irradiated sample of GrsA + TycB1(S5BzF) with different molecular markers (M1 and M2). h = high band, m = middle band, l = low band. Calculated masses are 132.5 kDa (GrsA) and 125.2 kDa (TycB1).

We incorporated the photo-crosslinking amino acid **BzF** into the first 13 positions (S2 to A14) of the acceptor module TycB1. These residues represent the “thumb” and the first β sheet of the “palm”-motif in the helix-hand model (Fig. 1D).^22^ M12 is the central hydrophobic amino acid in the β sheet that faces the donor-COM helix according to our proposed model.^22^ To monitor the crosslinking ability of all these constructs, each TycB1(BzF) protein was UV-irradiated either in the absence or presence of the GrsA partner protein and then analyzed by SDS-PAGE and immunoblotting against the SBP-tag on GrsA (Fig. 3A). Control experiments with wildtype TycB1 lacking **BzF** showed that no new bands were produced by UV-irradition (Fig. 3B), whereas most TycB1(BzF) constructs formed new bands even in the absence of GrsA (except those with D11BzF and Y13BzF), suggesting various forms of intra- or intermolecular crosslinking (see exemplary Fig. 3C and all lanes without GrsA in Fig. 3A). Depending on the protein batch, additional GrsA-independent bands as photo-crosslink products could become more pronounced, possibly through partially misfolded TycB1 species. Further control experiments exploring varying protein concentrations, irradiation times and buffer conditions were performed and showed that the observed crosslinks, which are discussed in the following, were reproducible over a wide range of conditions (Fig. S1–S3†).

In total, three types of new bands with very different migration behavior and apparent molecular weights exceeding those of the individual GrsA (132.5 kDa) and TycB1 (125.2 kDa) proteins became visible. We refer to these as low (l), middle (m) and high (h) bands (Fig. 3A). The low bands (∼160 kDa), when present, always appeared also in the absence of GrsA and did not stain in the GrsA-specific anti-SBP immunoblot. Its migration behavior corresponded to a molecular weight clearly below the calculated size of two TycB1 molecules. Together, these findings suggested the low bands represented (a) monomeric form(s) of TycB1 with an intramolecular crosslink. The medium bands migrated at >200 kDa, which potentially fitted with the calculated molecular weight of both the crosslinked GrsA–TycB1 heterodimer (257.7 kDa) and a TycB1–TycB1 homodimer (250.4 kDa). It could be observed without or with GrsA, but was more pronounced in its presence, and it stained in the SBP immunoblot in the latter cases. These findings suggested that the middle bands represented a form of a TycB1–TycB1 homodimer in samples lacking GrsA, and additionally a GrsA–TycB1 heterodimer in samples that included GrsA. Finally, the high bands were only observed in presence of GrsA and only for the V3, F4, S5, E7, Q8 and V9 positions of the TycB1(BzF) mutants (see Fig. S4† for densitometric analysis of band intensities), suggesting they represented GrsA–TycB1 hetero-crosslinked species. The finding that the high bands always stained in the SBP immunoblot is consistent with this interpretation.

Control experiments with gradually truncated COM domains on GrsA or TycB1 confirmed that the appearance of the GrsA-dependent high and middle bands was dependent on the intact COM regions and became weaker with their gradual deletion (Fig. S5†).

Since the migration behavior of these bands was difficult to determine precisely on our standard acrylamide Tris–glycine gels (6%) with a standard molecular weight marker (highest marker band at 200 kDa) as shown in Fig. 3A, we turned to a Tris–acetate gel (6%) using a special high-molecular weight marker (Fig. 3D). Using TycB1(S5BzF) as one example that showed all three bands, this analysis suggested the high band migrated well beyond 300 kDa (at ∼400 kDa). The middle band was determined more accurately to run at approx. 270–280 kDa and the low band migrated at 130–140 kDa (Fig. 3D). The calculated 257.7 kDa of a GrsA–TycB1 crosslink are thus best fitting to the middle band. The middle band is also similar in size to crosslinks previously obtained using GrsA(BzF) with **BzF** in the donor COM domain.^22^

The finding that the presence and intensity of the photo-crosslink products were clearly dependent on the **BzF** position (Fig. 3A and S4†), suggested that structural information on the architecture of the interface could be derived from this data.

### Branching pattern of crosslink impacts migration behavior in gel electrophoresis: nature of the L- and T-forms

**Fig. 4:**
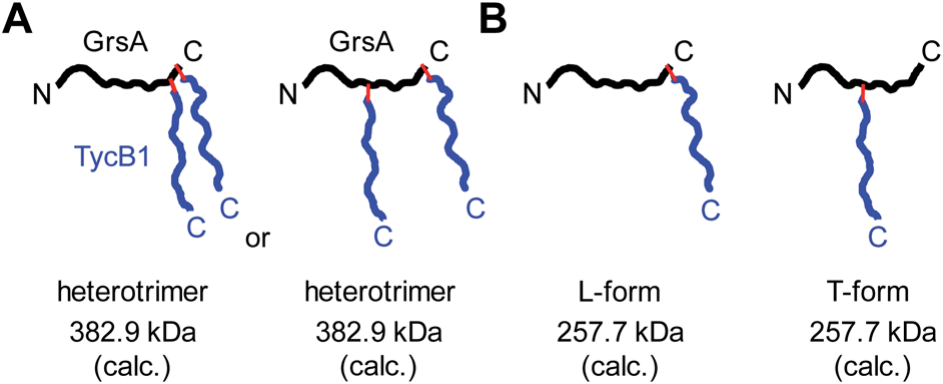
Models to explain the observed high band with aberrant migration behavior in SDS-PAGE gels. GrsA is represented as black line, TycB1 as blue line and red lines indicate covalent crosslinks. (A) Heterotrimer formation resulting from various forms of double crosslinks. (B) L- and T-form isomers with the T-form backbone connectivity causing unusual migration.

Why were there two apparently different crosslinks between TycB1 and GrsA in the middle and high bands? And how can the surprising high band with an apparent mass of ∼400 kDa be explained? As it significantly exceeds the calculated mass of the GrsA–TycB1 heterodimer, we hypothesized on two possible explanations for its formation. First, as illustrated in Fig. 4A, the high band could represent a trimer consisting of one molecule GrsA and two molecules of TycB1 (calculated molecular weight 382.9 kDa). Second, the high band would correspond to a GrsA–TycB1 heterodimer, however, the nature of the crosslink position influences the migration behavior in polyacrylamide gels such that it can become radically different from its calculated mass. Such an effect on electrophoretic migration behavior was shown when comparing linear and branched polyethylene glycol chains and was hypothesized to be the underlying cause of a protein post-translationally modified with SUMO in a central region.^42,43^ In this second scenario, we conceived two possible types of crosslink patterns, in which the **BzF**, located N-terminally in the TycB1 polypeptide chain, gave rise to covalent bonds either at a terminal or at an internal position in the GrsA polypeptide chain. We refer to these two crosslink isoforms as L- and T-forms, respectively (Fig. 4B).

**Fig. 5:**
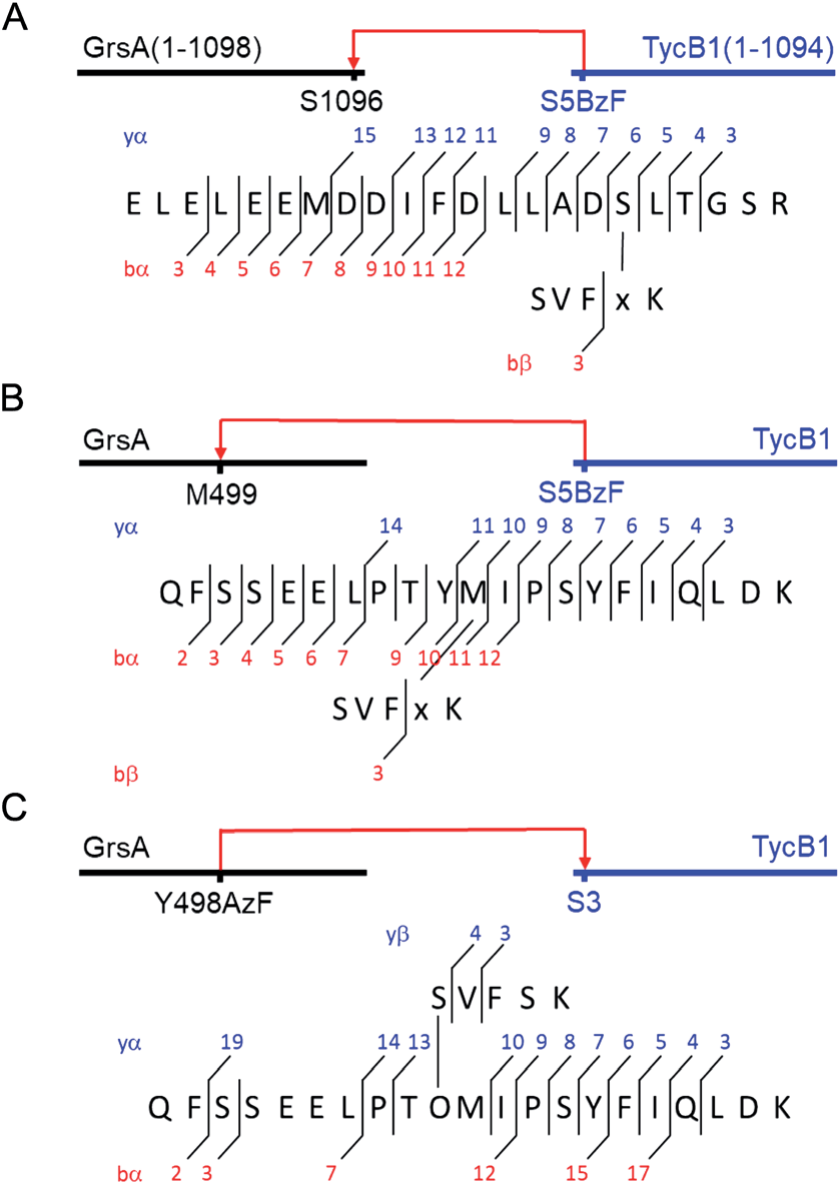
MS/MS mapping analysis of photo-crosslinked peptides. The MS/MS spectra are consistent with the expected fragmentation patterns of the illustrated crosslinked peptides, identified using StavroX 3.6.6.^44^ (A) Assignment of a cross-link between GrsA (α) and TycB1(S5BzF) (β) peptides from the middle band of the photo reaction x denotes **BzF**. The precursor ion [M + 4H]^4+^ at *m*/*z* 811.1434 matches the expected mass of the cross-linked peptide with a deviation of 0.7 ppm. The GrsA fragment encompasses amino acids E^1080^LELEEMDDIFDLLADSLT^1098^ and the additional residues GSR from the fused tag. (B) Assignment of a crosslink between GrsA (α) and TycB1(S5BzF) (β) peptides from the high band of the photo reaction x denotes **BzF**. The precursor ion [M + 4H]^4+^ at *m*/*z* 817.4054 matches the expected mass of the cross-linked peptide with a deviation of −0.3 ppm. The GrsA fragment encompasses amino acids Q^489^FSSEELPTYMIPSYFIQLDK^509^. (C) Assignment of a cross-link between GrsA(Y498AzF) (α) and TycB1 (β) peptides from the high band of the photo-reaction. O denotes **AzF**. The precursor ion [M + 4H]^4+^ at *m*/*z* 779.6391 matches the expected mass of the cross-linked peptide with a deviation of 0.4 ppm. The TycB1 fragment encompasses amino acids S^2^VFSK.^6^ The associated MS/MS spectra are presented in the ESI (Fig. S6 and S11†).

To probe the two different models, we mapped the crosslink positions by tandem mass spectrometry (MS/MS). The middle and high bands of a photo-crosslink experiment using GrsA and TycB1(S5BzF) were excised from the SDS gel, digested with trypsin and analyzed by LC-MS/MS. In the middle band digest, at least two chromatographically distinct isobaric peptides with *m*/*z* 811.14 were identified, both corresponding to crosslinks to the GrsA donor COM helix (downstream of E1080). In one case, the fragmentation data quality allowed us to pin down the crosslink site to S1096 (Fig. 5A and S6A†), whereas for the second peptide, either I1089 or F1090 is the target (Fig. S6B†). Close proximity of these residues with S5 in the acceptor COM domain is consistent with our structural helix-hand model of the COM domain interface.^22^ According to our second hypothesis, the resulting shape of these crosslinked GrsA–TycB1 species would resemble the L-form with a terminus-to-terminus crosslink (Fig. 4B).

Interestingly, the crosslinks identified in the high band mapped to a markedly different position. The amino acid stretch P^496^TYMI^500^ of GrsA was recovered with M499 as the crosslink site (Fig. 5B and S6C†). Surprisingly, this internal crosslink site is located outside of the terminally located COM-interface (compare Fig. 1C for GrsA numbering). The biochemical conclusions from this finding are discussed below. Notably, the crosslinking to this interior position of GrsA would result in the T-form shape of two polypeptides, as postulated in our second hypothesis (Fig. 4B). Similar results were obtained for the middle and high bands of a photo-crosslink experiment using GrsA and TycB1(V3BzF) (Fig. S7†).

To rule out the possibility of an artefactual nature of the identified crosslinks, which might be conceivable due to the non-native pairing of GrsA with TycB1, we also analyzed photo-crosslink products in all possible combinations of the first two modules of the gramicidin S and tyrocidine synthetases. **BzF** was incorporated at the corresponding position of the acceptor COM domain of GrsB1 (K5BzF). Indeed, the bands representing the L- and T-form crosslinks were observed in all native and non-native combinations, however, with varying relative intensities (Fig. S8 and S9†). TycB1(S5BzF) was more prone to the formation of the T-form crosslink, both with GrsA and with its native partner TycA. On the other hand, GrsB1(K5BzF) resulted mostly in formation of the L-form crosslink with both protein partners, but also the T-form crosslink with the internal position could be mapped (Fig. S9†). A TycA–TycB1 fusion construct as a control migrated similar to middle bands, thus providing further confirmation for their assignment as L-form isomers (Fig. S8†).

While these results supported the second hypothesis to explain the middle and high bands as L-form and T-form crosslink products (Fig. 4B), they did not strictly rule out the first hypothesis because a second crosslink leading to a potential GrsA–(TycB1)_2_ heterotrimer might have escaped the detection. However, since the terminus–terminus crosslinks (L-form) were exclusively found in the middle bands and the terminus-internal crosslinks (T-form) exclusively in the high bands, the heterotrimer model of the first hypothesis appeared very unlikely (Fig. 4A). Nevertheless, given the difficulty to prove the absence of a possible heterotrimeric reaction product, which would be necessary to disprove the first hypothesis, we aimed at collecting direct evidence to prove the second hypothesis.

### Preparation of defined L- and T-form conjugates by CuAAC and the reverse photo-crosslinking experiment prove the high band to be the T-form

**Fig. 6:**
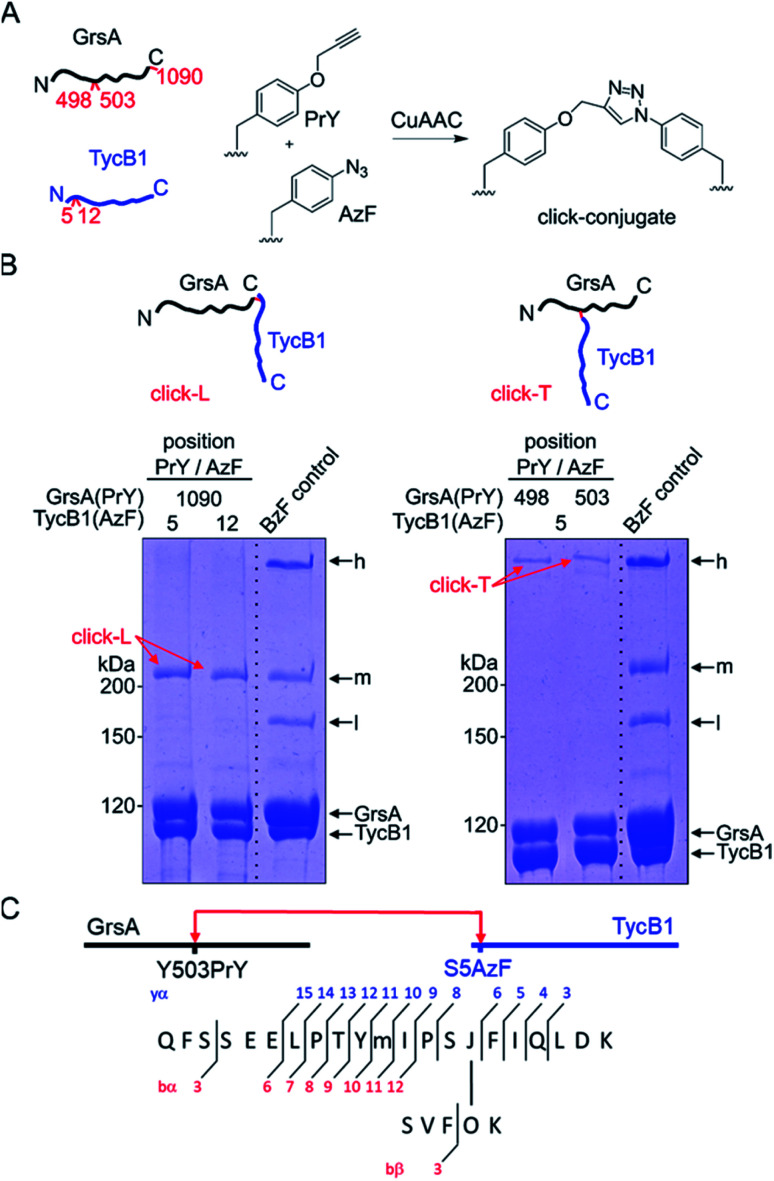
CuAAC-mediated synthesis of standards of L- and T-form isomers. (A) Scheme of the reactions with **AzF** and **PrY** at the indicated positions. (B) Synthesized click-L and click-T isoforms are illustrated. Analysis of the CuAAC reactions by coomassie-stained SDS-PAGE gels is shown with a photo-crosslink reaction of TycB1(S5BzF) with GrsA for comparison (**BzF** control). (C) Assignment of a cross-link between GrsA(Y503PrY) (α) and TycB1(S5AzF) (β) peptides from the high band of the CuAAC reaction. O and J denote **AzF** and **PrY**, respectively. The precursor ion [M + 4H]^4+^ at *m*/*z* 815.1499 matches the expected mass of the cross-linked peptide with a deviation of −2.7 ppm, and the MS/MS spectrum is consistent with the expected fragmentation pattern. The associated MS/MS spectra of this click-T standard as well as the other synthesized click-L and click-T standards are presented in Fig. S10.†

We decided to synthesize defined covalent conjugates of the GrsA and TycB1 proteins at the crosslink positions identified in the middle and high bands to evaluate their migration behavior as references for the L- and T-form species on SDS-PAGE gels. In order to perform conjugate formation by bioorthogonal copper-catalyzed alkyne–azide cycloaddition (CuAAC; “click” reaction),^45,46^ we incorporated the unnatural amino acids *p*-propargyloxyphenylalanine (**PrY**)^47,48^ and *p*-azidophenylalanine (**AzF**)^41^ at interior (Y498, Y503) and terminal (F1090) positions in GrsA, as well as at terminal positions (S5, M12) in TycB1 (Fig. 6A). To generate triazole-linked GrsA–TycB1 heterodimers in a terminus–terminus connectivity as the L-form reference, GrsA(F1090PrY) was thus mixed with either TycB1(S5AzF) or TycB1(M12AzF) in the presence of the copper catalyst. SDS-PAGE analysis showed that the click product (click-L) co-migrated with the middle band obtained from the GrsA/TycB1(S5BzF) photo-crosslinking reaction, consistent with the calculated size of the heterodimer (Fig. 6B, left panel). Next, to generate GrsA–TycB1 heterodimers linked through internal and terminal positions as the T-form reference, the CuAAC reaction was performed with GrsA(Y498PrY) or GrsA(Y503PrY) mixed with partner protein TycB1(S5AzF). Strikingly, this experiment yielded triazole-linked conjugates (click-T) that indeed co-migrated with the high band of the standard of the photo-crosslinking reaction (Fig. 6B, right panel). The low yields observed for the CuAAC-mediated conjugations can likely be attributed to the enormous sizes of the two proteins and a denaturing effect of the catalyst on the folding of the proteins. The formation of protein–protein conjugates by CuAAC typically results in incomplete reactions.^49–51^ The proper triazol-linkages in the GrsA–TycB1 conjugates were verified by LC-MS/MS (Fig. 6C and S10†). Together, these findings provided clear and direct evidence for our second hypothesis as they explained the migration behavior of the middle and high bands of the photo-crosslinking experiments as L- and T-form isomers of the GrsA–TycB1 heterodimers (Fig. 4B).

To further validate the unexpected spatial proximity suggested by the T-form crosslinks we asked whether the proximity could also be observed in a ‘reverse’ photo-crosslinking experiment. To this end, photo-crosslinking amino acids **BzF** and **AzF** were incorporated at position Y498 of GrsA right next to M499 that was identified by MS-mapping. Indeed, following incubation of GrsA(Y498AzF) with TycB1 and UV-irradiation we mapped the crosslink to peptide S^2^VFSK^6^ of TycB1 (Fig. 5C and S11†). These findings independently confirmed the spatial proximity of the terminal TycB1 region and the internal GrsA regions. They also further supported the notion that the T-form crosslink did not result from potential structural artefacts caused by the unnatural amino acid **BzF** or the preference of **BzF** for crosslinking with methionine residues.^38^

### Implications of the identified crosslinks on the domain architecture in the GrsA–TycB1 system

Both L-form and T-form crosslinks provided important distance information to understand the NRPS three-dimensional domain arrangements and superstructure. We will first discuss the unexpected crosslinks that resulted in the T-form isomers. These findings established a spatial proximity between the residues of the N-terminal acceptor COM domain of TycB1 and the internal peptide sequence PTYMI of GrsA (aa496–500), and in particular of M499. This latter region is located in the small and mobile A^C^ subdomain of the bilobed A domain (compare Fig. 1C for domain organization),^52^ thus outside the assumed COM domain interaction interface. It is part of a typically conserved region, which was previously designated as the A9 core motif of adenylation domains.^1^ No particular function has been ascribed to the A9 core motif. It constitutes a structurally conserved turn-helix element in an exposed position of the A^C^ subdomain.^52^ The A^C^ domain of a module preceding a COM interaction pair is not adjacent to the COM acceptor domain on the level of the primary sequence (Fig. 1C), suggesting that the revealed spatial proximity must be brought about through the three-dimensional domain arrangement. There is no known functional relation for this interaction up to now and our data only establishes the proximity between these two regions without an indication for a functional relevance. Importantly, however, when such contacts are observed in protein crystallography^18^ it is difficult to tell whether they were artificially enforced by crystal packing effects. In contrast, our findings resulted from experiments performed in solution under conditions compatible with catalysis and therefore lend more credibility to the notion that the enzymes were captured in a native and functionally relevant conformation.

**Fig. 7:**
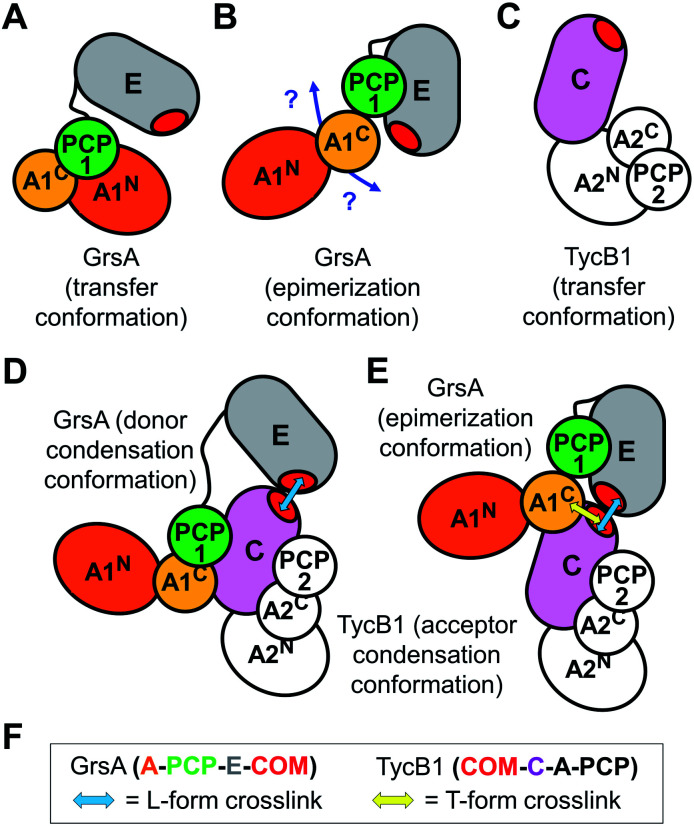
Illustration of possible intra- and inter-module conformations. (A and C) In the transfer conformation the PCP interacts with the A domain. The localization of the E domain is unknown. (B) In the epimerization conformation the PCP interacts with the E domain and the localization of the A domain is unknown. (D) In the donor condensation conformation the PCP is interacts with the donor position of the C domain. (E) In the acceptor condensation conformation the PCP interacts with the acceptor position of the C domain. The COM domains are essential to facilitate efficient intermodule interactions as shown in (D and E). Blue and yellow arrows indicate the proximities confirmed by photo-crosslinking, giving rise to L- and T-form crosslinks, respectively. (F) Graphical legend.

To rationalize the captured proximity between the A^C^ domain of GrsA and the acceptor COM domain of TycB1 we attempted to conclude on the most likely underlying conformation of the GrsA–TycB1 complex. Notably, this endeavor is complicated by the fact that neither structures of the donor COM domain or a native COM domain complex nor of a PCP-E-C sequence of domains are available. The E domain of GrsA is an additional binding site for the PCP that is not represented in known structures of entire NRPS modules. We reasoned that the A^C^ subdomain of GrsA will be partially dragged on the backside of the PCP to the catalytic domains, as observed in several crystal structures.^17,18^ Next to possible open structures with the PCP not being in functional domain contact, the three expected positions the PCP can adopt are those in contact with the catalytic centers of the A and E domains of GrsA and the C domain of TycB1. We term the respective conformations as transfer, epimerization and donor condensation conformations (Fig. 7A, B, and D). The PCP of the TycB1 module could adopt transfer and acceptor condensation conformations (Fig. 7C–E). Crystal structures from other NRPS systems are known for the transfer^9,13,14,18,19^ and the donor condensation conformations.^9^ Despite the lack of the E domain in these structures, we hypothesized that they would allow us to estimate whether the relative orientation and distance of the A9 motif in the A^C^ subdomain to the other domains would be compatible with the proximity of the acceptor COM observed in this study. Interestingly, crystal structures of both the transfer and the donor condensation conformations showed the sequence corresponding to the P^496^TYMI^500^ sequence of the GrsA A9 motif to be at the center of the domain contacts with the PCP and the respective catalytic domain, A^N^ or C (Fig. 7A and D). These arrangements result in the A9 motif being almost completely engulfed and therefore very likely not available for any further domain contacts with the COM domain (Fig. S12A and B†). Furthermore, the obviously conserved structural role of the A9 motif in domain contacts is also found when the A^C^-PCP unit binds to the C domain in the *acceptor* position (illustrated in Fig. 7D and E for TycB1, not shown in detail).^18,19^ While these observations support the propensity of the A9 motif for contacts with other domains, they appear to rule out the transfer and donor condensation conformations as the conceivable domain constellations for the proximity with the COM domain. Another argument against the donor condensation conformation can be construed from the estimated location of the photo-crosslinking side chain in the COM domain complex, which would be ∼44 to 55 Å away from the A9 motif, on the opposite side of the C-domain (illustrated in Fig. 7D).

We therefore attempted to evaluate whether the unknown epimerization conformation could be compatible with a close proximity between the acceptor COM domain of TycB1 and the A9 motif of the GrsA A^C^ subdomain. An isolated PCP-E structure is known (pdb: 5ISX)^16^. To project possible localizations of the A^C^ domain relative to the PCP-E ensemble (Fig. 7B) we overlayed the PCP-E structure with A^C^-PCP units from several other structures. This modeling suggested that the contact between the A^C^ subdomain and the acceptor COM domain in three-dimensional space is plausible with the PCP binding the E domain (Fig. S12C†), although these findings do not provide a solid proof. Together, we assume that the mapped crosslink of the T-form isomer most likely captured the GrsA–TycB1 complex in the epimerization conformation as illustrated in Fig. 7E.

Furthermore, all **BzF** positions that gave rise to T-form crosslinks were in the ‘thumb’ region of the hand motif^17,22^ from aa3–9 (Fig. 3 and S4†). A protrusion of the thumb away from the compactly folded C-domain,^17^ as observed in pdb-file 2VSQ, may explain why it can be in contact with both the donor COM helix and the unrelated A^C^ domain. The similarly observed L-form crosslinks from the ‘thumb’ positions reflect the simultaneous interaction in the COM–COM pair.

**BzF** positions at aa10–14 of TycB1 are located in the first β sheet of the ‘palm’ in the hand motif. The residues of the β sheet facing the one side are expected to be completely covered when binding the donor COM helix. Consequently, only L-form crosslinks with the COM donor domain were observed. The finding that D11BzF and Y13BzF failed to produce crosslinks, whereas Q10BzF, M12BzF and A14BzF did, is consistent with the alternating orientation of these side chains in the β sheet such that only every second residue would face the helix of the COM donor motif and the others are turned towards the interior of the C domain. Importantly, these results show in fact the first direct proof for residues of the ‘palm’ region to be involved in the COM–COM interaction and thus further strengthen the helix-hand model. A more comprehensive photo-crosslinker-scanning and crosslink mapping analysis will therefore likely reveal a more detailed view on the COM domain structure.

### T-form crosslinks to the non-neighboring A^C^ domain are sensitive to substrate-induced conformational changes

**Fig. 8:**
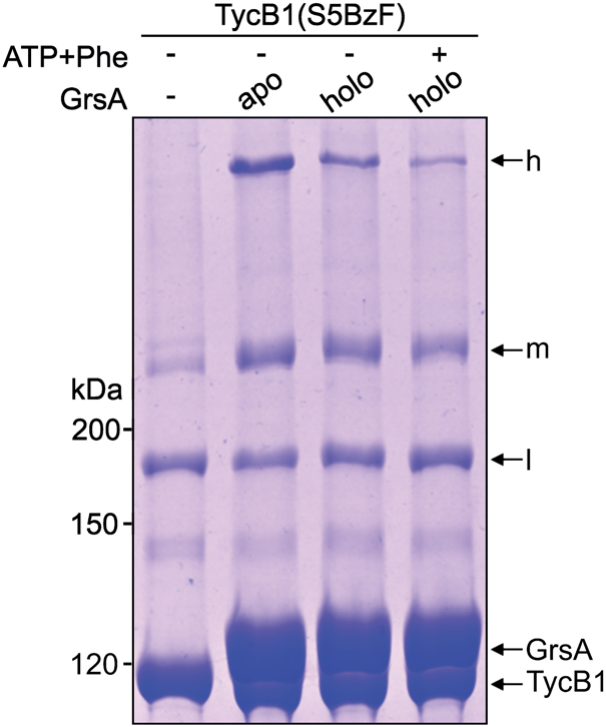
Crosslink assay of TycB1(S5BzF) with the cognate partner protein GrsA in different chemical states. Shown is a coomassie-stained SDS-PAGE gel. Before incubation with TycB1(S5BzF) for 1 h at 37 °C and UV-irradiation (*λ* = 366 nm) for 1 h, GrsA was preincubated with 2 mM ATP and 1 mM L-Phe for 10 minutes at room temperature. h = high band, m = middle band, l = low band. Calculated masses are 132.5 kDa (GrsA) and 125.2 kDa (TycB1).

Since NRPS conformations can be shifted between different equilibria depending on the catalytic state of the protein,^20,53^ we finally asked if the conformation that gave rise to the T-form crosslink between the acceptor COM domain and the A^C^ subdomain can be favored or disfavored under different enzymatic conditions, reflected by an increased or decreased intensity of the crosslink band, respectively. To this end, we performed the photo-crosslinking experiment of TycB1(S5BzF) with GrsA in three different chemical states. Fig. 8 shows that indeed clear differences in the relative intensities of the crosslink bands were observed, with less T-form product being produced with the 4′-phosphopantetheinylated holo-form of GrsA compared to its apo-from, and a further reduction observed with aminoacylated holo-GrsA, formed in the presence of ATP and L-Phe. In contrast, the intensity of the L-form isomer remained unchanged, supporting the notion that the interaction mediated through the donor and acceptor COM domains is unaffected, which is in agreement with our previous study.^22^ These results are indeed consistent with our conclusion that the epimerization conformation of GrsA is likely the relevant conformation for the T-form crosslink (see above), because in a previous investigation of domain conformations in solution we found that the formation of the aminoacyl-thioester favors back-binding of the PCP to the A domain to adopt a post-transfer conformation.^20,53^ Accordingly, upon addition of substrates, an equilibrium of possible conformations in GrsA should be shifted in disfavor of the epimerization conformation, in agreement with the observed decrease of T-form crosslink efficiency. Interestingly, these findings also suggest that photo-crosslinkers might be useful tools to address conformational dynamics in NRPSs.

## Conclusions

In conclusion, we could show that a T-shaped crosslink of two polypeptide chains can have a completely different migration behavior on an SDS gel than its isomer crosslinked in L-form. To our knowledge, this is the first systematic study to investigate and directly prove such kinds of protein backbone isomers. Our insights on L- and T-form crosslinks should also be applicable to other types of branched polypeptide chains, such as in proteins posttranslationally modified with ubiquitin-like proteins^43,54^ or in branched intermediates of intein-mediated protein splicing pathways.^55,56^ We suspect the lengths of the polypeptide chains originating from the branch point as well as the pore size of the polyacrylamide gels to be the decisive parameters for whether or not such isomers exhibit sharply differing migration behaviors in gel electrophoresis.

By photo-crosslinking and peptide mapping we have shown that a functionally unrelated and in primary sequence non-neighboring A^C^ domain of the NRPS template can be localized in spatial proximity to the interaction mediating COM domain interface of two subunits. To our knowledge, this is the first non-neighboring domain contact in 3-D space unraveled for NRPSs in solution. Our results suggest a rational approach to investigate the three dimensional packing of domains in multimodular NRPS on the molecular level by photo-crosslinking to unravel their higher-order architecture, which is mostly uncharted territory.

## Experimental methods

### General

Restriction enzymes were from Thermo Scientific, buffer reagents, antibiotics and media components were from Carl Roth. *para*-l-Benzoyl-phenylalanine (**BzF**) and *para*-azido-l-phenylalanine (**AzF**) were from Bachem, propargyl-l-tyrosine (**PrY**) was from Iris Biotech. Solvents for HPLC were from VWR. PerfectoPro Ni-NTA agarose was from 5 PRIME and streptactin sepharose as well as desthiobiotin were from IBA. Antibodies for western blot analysis were from Roche (anti-His), and secondary antibodies were from GE Healthcare. Synthetic oligonucleotides were from Biolegio.

### Protein preparation

Recombinant proteins were expressed and purified using Ni-NTA or streptavidin affinity chromatography as previously reported.^22^ Unnatural amino acids (**BzF**, **AzF** and **PrY**) were incorporated using nonsense suppression. For **BzF** and **AzF** the corresponding pEVOL plasmids and for **PrY** the plasmid pUltra-CNF were introduced into *E. coli* expression strains.^39,41,48,57^ Each Uaa was added in a final concentration of 1 mM to the growth medium. All assays of the proteins were performed in NRPS buffer (50 mM HEPES, 100 mM NaCl, 10 mM MgCl_2_, 1 mM EDTA, pH 7.0) unless differently specified. For storage at −80 °C, proteins were flash–frozen in liquid nitrogen in presence of 10% glycerol (v/v).

### Microscale thermophoresis (MST)

MST measurements were performed with the Microscale Thermophoresis Monolith NT.115 (NanoTemper, Germany). GrsA was fluorescently labeled using the Labeling Kit RED-NHS (L001, NanoTemper, Germany). The concentration of fluorescently labeled GrsA was 1 nM and TycB1 was added in a dilution series in NRPS buffer with 0.05% (v/v) Tween-20 as indicated. The intensity of the laser excitation (LED power) was adjusted to give fluorescence intensity values between 400 and 1200 counts. The intensity of the MST excitation (MST-Power) varied between 20 and 40%. The binding was quantified from the ratio of *F*_hot_ (fluorescence after thermal diffusion) to *F*_cold_ (initial fluorescence). For evaluation, the change in fluorescence was plotted against the decadic logarithmic concentration of the interaction partner in the dilution series. The *K*_d_ value was calculated from the data of the MST curves as sigmoidal fit using the software NT Analysis (NanoTemper, Munich).

### Crosslinking assay

Proteins (each 5 μM) were pre-incubated at 25 °C for 45 min and then irradiated with UV light (*λ* = 366 nm, 8 W, 1.5 cm distance to the UV lamp) for 45 min. All samples were analyzed by SDS-PAGE and visualized by Coomassie staining and western blot.

### Cu(i)-catalyzed azide–alkyne cycloaddition

Azide- and alkyne-functionalyzed proteins (each 4 μM) were incubated in the presence of 1 mM TCEP, 100 μM TBTA and 500 μM CuSO_4_ for 30 min at 25 °C.

### In-gel tryptic digest followed by LC-MS/MS analysis of proteins

SDS-PAGE bands of crosslink products were excised, destained, and proteins were reduced with 10 mM DTT, alkylated with 55 mM iodoacetamide, and digested with 400 ng trypsin at 37 °C for 2 h in the presence of ProteaseMax (Promega Corp., Madison, WI, USA) according to the manufacturer instructions. The acidified supernatant (addition of formic acid to achieve pH 2–3) was then analyzed on an LC-MS consisting of an UltiMate™ 3000 RS LC nano system (Thermo Fisher Scientific GmbH, Dreieich, Germany) connected to a maXis II UHR-TOF mass spectrometer with a CaptiveSpray nano-ESI source (Bruker Daltonik GmbH, Bremen, Germany) equipped with a nanoBooster device. For all proteins, 10 μL of the digest solution were injected. The solution was loaded on a C18 trapping column (Acclaim PepMap 100, 5 μm, 100 Å, ID 300 μm × L 5 mm, Thermo Scientific) at a flow rate of 20 μL min^−1^ in 2% eluent B (eluent A: 0.1% formic acid in water; eluent B: 0.1% formic acid in acetonitrile). At the same time, a solution of sodium formate clusters in 50% 2-propanol was injected to enable post-run calibration of MS and MS/MS spectra. After 10 min of washing at 2% B, a 50 min gradient (2% to 60% B, flow rate 300 nL min^−1^) was applied for the separation on a C18 nano column (Acclaim PepMap 100, C18, 2 μm, 100 Å, ID 0.075 mm × L 250 mm, Thermo Fisher Scientific GmbH, Dreieich, Germany). MS settings: capillary voltage 1.200 V, mass range: *m*/*z* 150-2200. MS survey scans were performed with a cycle time of 3 s. After each survey scan, the 10 to 20 most abundant precursor ions with *z* > 1 were selected for fragmentation using collision-induced dissociation. MS/MS summation time was adjusted depending on the precursor intensity. The applied precursor isolation window and the collision energy were chosen depending on the precursor *m*/*z* and charge.

DataAnalysis 4.4 (Bruker Daltonik GmbH, Bremen, Germany) was used for chromatogram processing and ProteinScape 4.0.3 (Bruker Daltonik GmbH, Bremen, Germany) was used to search our in-house database and for further analysis of MSMS data. Crosslink peptides were identified using StavroX 3.6.6 (Michael Götze, University of Halle-Wittenberg).^44^

## Conflicts of interest

There are no conflicts to declare.

## Supplementary Material

SC-011-D0SC01969K-s001
