# Supplementary material for: Photo-crosslink analysis in nonribosomal peptide synthetases reveals aberrant gel migration of branched crosslink isomers and spatial proximity between non-neighboring domains
Source: Chem Sci. 2020 Aug 11;11(33):8945–54. doi: 10.1039/d0sc01969k (PMC8163358; doi:10.1039/d0sc01969k)
Supplement: SC-011-D0SC01969K-s001 [file SC-011-D0SC01969K-s001.pdf]

Electronic Supplementary Material (ESI) for Chemical Science.
This journal is © The Royal Society of Chemistry 2020

ELECTRONIC SUPPLEMENTARY INFORMATION

Photo-crosslink analysis in nonribosomal peptide synthetases reveals aberrant gel migration of branched crosslink isomers and spatial proximity between non-neighboring domains

Eva Dehling,# Jennifer Rüschenbaum,# Julia Diecker, Wolfgang Dörner, Henning D. Mootz*

Institute of Biochemistry, Department of Chemistry and Pharmacy, University of Muenster, D-48149 Münster, Germany

* corresponding author, e-mail: Henning.Mootz@uni-muenster.de

# = equally contributed

Table S1 page S2
Table S2 S2
Table S3 S3
Figure S1 S6
Figure S2 S6
Figure S3 S7
Figure S4 S7
Figure S5 S8
Figure S6 S9
Figure S7 S11
Figure S8 S12
Figure S9 S13
Figure S10 S14
Figure S11 S16
Figure S12 S17
Electronic Supplementary References S18

SUPPLEMENTARY TABLES

Table S1. Information on pdb files used to generate Figures S12C.

Pdb code	Ref	NRPS	Domain organization	Conformation A domain	Localization PCP domain	Color A^C subdomain
4ZXI	[1]	AB3403	C-A-PCP-TE	A conformation	C domain acceptor position	Salmon
4ZXH	[1]	AB3403	C-A-PCP-TE	A conformation	C domain acceptor position	Deep salmon
5ES9	[2]	LgrA	F-A-PCP	Stretched open A conformation	F domain	Warm pink
2VSQ	[3]	SrfA-C	C-A-PCP-TE	Stretched open A conformation	apo-PCP at C domain acceptor position	Dark salmon
6N8E	[4]	ObiF1	C-A-PCP-TE-MLP	A conformation	C domain acceptor position, with TE domain	Raspberry
6MFX	[5]	LgrA	F-A-PCP-C-A	A conformation	C domain donor position	Brown
6MFY	[5]	LgrA	F-A-PCP-C-A	Unusal conformation	C domain donor position	TV red
6MFZ	[5]	LgrA	F-A-PCP1-C-A-PCP2	A conformation	PCP1 at C domain donor position	Firebrick
6MFW	[5]	LgrA	F-A-PCP-C	A conformation	C domain donor position	Red

Table S2. List of recombinantly produced proteins used in this study and their encoding plasmids.

Name of construct*	Encoding plasmid	Vector backbone
[TycB1] C(S2X)-A-PCP-His_6	pED42	pTrc99a
[TycB1] C(V3X)-A-PCP-His_6	pED43	pTrc99a
[TycB1] C(F4X)-A-PCP-His_6	pED44	pTrc99a
[TycB1] C(S5X)-A-PCP-His_6	pED45	pTrc99a
[TycB1] C(K6X)-A-PCP-His_6	pED46	pTrc99a
[TycB1] C(E7X)-A-PCP-His_6	pED31	pTrc99a
[TycB1] C(Q8X)-A-PCP-His_6	pED47	pTrc99a
[TycB1] C(V9X)-A-PCP-His_6	pED50	pTrc99a
[TycB1] C(Q10X)-A-PCP-His_6	pED48	pTrc99a

[TycB1] C(D11X)-A-PCP-His_6	pED39	pTrc99a
[TycB1] C(M12X)-A-PCP-His_6	pED40	pTrc99a
[TycB1] C(Y13X)-A-PCP-His_6	pED49	pTrc99a
[TycB1] C(A14X)-A-PCP-His_6	pED87	pTrc99a
[TycB1] C-A-PCP-His_6	ProCAT_EDnat	pTrc99a
[GrsA] SBP-A-PCP-E-His_6	pGV196	pET28a
[GrsA] SBP-A-PCP-E(Y498X)-His_6	pED134	pET28a
[GrsA] SBP-A-PCP-E(Y503X)-His_6	pED135	pET28a
SBP-[GrsA] A-PCP-E(F1090X)-His_6	pGV176	pET28a
[GrsB1] C(K5X)-A-PCP-His_6	pJR73	pTrc99a
[TycA] SBP-A-PCP-E-His_6	pJR88	pET28a
[TycA-TycB] SBP-A-PCP-E--C-A-PCP-His_6	pJR96	pET28a

* X denotes amino acid incorporation in response to amber stop codon.

Table S3. Sequences of recombinantly produced proteins

**Protein**	**Sequence**
[GrsA] SBP-A-PCP-E-His_6	MDEKTTGWRGGHVVEGLAGELEQLRARLEHHPQGQREPGASMLNSSK SILIHAQNKNGTHEEEQYLFAVNNTKAEYPRDKTIHQLFEEQVSKRPNN VAIVCENEQLTYHELNVKANQLARIFIEKGIGKDTLVGIMMEKSIDLFIGI LAVLKAGGAYVPIDIEYPKERIQYILDDSQARMLLTQKHLVHLIHNIQFN GQVEIFEEDTIKIREGTNLHVPSKSTDLAYVIYTSGTTGNPKGTMLEHKG ISNLKVFFENSLNVTEKDRIGQFASISFDASVWEMFMALLTGASLYIILK DTINDFVKFEQYINQKEITVITLPPTYVVHLDPERILSIQTLITAGSATSPSL VNKWKEKVTYINAYGPTETTICATTWVATKETIGHSVPIGAPIQNTQIYI VDENLQLKSVGEAGELCIGGEGLARGYWKRPELTSQKFVDNPFVPGEK LYKTGDQARWLSDGNIEYLGRIDNQVKIRGHRVELEEVESILLKHMYIS ETAVSVHKDHQEQPYLCAYFVSEKHIPLEQLRQFSSEELPTYMIPSYFIQ LDKMPLTSNGKIDRKQLPEPDLTFGMRVDYEAPRNEIEETLVTIWQDVL GIEKIGIKDNFYALGGDSIKAIQVAARLHSYQLKLETKDLLKYPTIDQLV HYIKDSKRRSEQGIVEGEIGLTPIQHWFFEQQFTNMHHWNQSYMLYRP NGFDKEILLRVFNKIVEHHDALRMIYKHHNGKIVQINRGLEGTLFDFYT FDLTANDNEQQVICEESARLQNSINLEVGPLVKIALFHTQNGDHLFMAI HHLVVDGISWRILFEDLATAYEQAMHQQTIALPEKTDSFKDWSIELEKY ANSELFLEEAEYWHHLNYYTENVQIKKDYVTMNNKQKNIRYVGMELT IEETEKLLKNVNKAYRTEINDILLTALGFALKEWADIDKIVINLEGHGRE EILEQMNIARTVGWFTSQYPVVLDMQKSDDLSYQIKLMKENLRRIPNK GIGYEIFKYLTTEYLRPVLPFTLKPEINFNYLGQFDTDVKTELFTRSPYSM GNSLGPDGKNNLSPEGESYFVLNINGFIEEGKLHITFSYNEQQYKEDTIQ QLSRSYKQHLLAIIEHCVQKEDTELTPSDFSFKELELEEMDDIFDLLADS LTGSRSHHHHHH
[GrsB1] C-A-PCP-His_6	MSTFKKEHVQDMYRLSPMQEGMLFHALLDKDKNAHLVQMSIAIEGIV DVELLSESLNILIDRYDVFRTTFLHEKIKQPLQVVLKERPVQLQFKDISSL DEEKREQAIEQYKYQDGETVFDLTRDPLMRVAIFQTGKVNYQMIWSFH HILMDGWCFNIIFNDLFNIYLSLKEKKPLQLEAVQPYKQFIKWLEKQDK QEALRYWKEHLMNYDQSVTLPKKKAAINNTTYEPAQFRFAFDKVLTQ

	QLLRIANQSQVTLNIVFQTIWGIVLQKYNSTNHVVYGSVVSGRPSEISGI<br>EKMVGLFINTLPLRIQTQKDQSFIELVKTVHQNVLFSQQHEYFPLYEIQN<br>HTELKQNLIDHIMVIENYPLVEELQKNSIMQKVGFTVRDVKMFEPTNYD<br>MTVMVLPRDEISVRLDYNAAVYDIDFIKKIEGHMKEVALCVANNPHVL<br>VQDVPLLTKQEKQHLLVELHDSITEYPDKTIHQLFTEQVEKTPEHVAVV<br>FEDEKVTYRELHERSNQLARFLREKGVKKESIIGIMMERSVEMIVGILGI<br>LKAGGAFVPIDPEYPKERIGYMLDSVRLVLTQRHLKDKFAFTKETIVIED<br>PSISHELTEEIDYINESEDLFYIIYTSGTTGKPKGVMLEHKNIVNLLHFTFE<br>KTNINFSDKVLQYTTCSFDVCYQEIFSTLLSGGQLYLIRKETQRDVEQLF<br>DLVKRENIEVLSFPVAFLKFIFNEREFINRFPTCVKHIITAGEQLVVNNEF<br>KRYLHEHNVHLHNHYGPSETHVVTTYTINPEAEIPELPPIGKPISNTWIYI<br>LDQEQQLQPQGIVGELYISGANVGRGYLNNQELTAEKFFADPFRPNER<br>MYRTGDLARWLPDGNIEFLGRADHQVKIRGHRIELGEIEAQLLNCKGV<br>KEAVVIDKADDKGGKYLCAYVVMEVEVNDSELREYLGKALPDYMIPS<br>FFVPLDQLPLTPNGKIDRKSLPNLEGIVNTNAKYVVPTNELEEKLAKIWE<br>EVLGISQIGIQDNFFSLGGHSLKAITLISRMNKECNVDIPLRLLFEAPTIQE<br>ISNYINGAKKESGSRSHHHHHH
[TycA] SBP-A-PCP-E-His$_6$	MDEKTTGWRGGHVVEGLAGELEQLRARLEHHPQGQREPMVANQANLI<br>DNKRELEQHALVPYAQGKSIHQLFEEQAEAFPDRVAIVFENRRLSYQEL<br>NRKANQLARALLEKGVQTDSIVGVMMEKSIENVIAILAVLKAGGAYVPI<br>DIEYPRDRIQYILQDSQTKIVLTQKSVSQLVHDVGYSGEVVVLDEEQLD<br>ARETANLHQPSKPTDLAYVIYTSGTTGKPKGTMLEHKGIANLQSFFQNS<br>FGVTEQDRIGLFASMSFDASVWEMFMALLSGASLYILSKQTIHDFAAFE<br>HYLSENELTIITLPPTYLTHLTPERITSLRIMITAGSASSAPLVNKWKDKL<br>RYINAYGPTETSICATIWEAPSNQLSVQSVPIGKPIQNTHIYIVNEDLQLL<br>PTGSEGELCIGGVGLARGYWNRPDLTAEKFVDNPFVPGEKMYRTGDLA<br>KWLTDGTIEFLGRIDHQVKIRGHRIELGEIESVLLAHEHITEAVVIAREDQ<br>HAGQYLCAYYISQQEATPAQLRDYAAQKLPAYMLPSYFVKLDKMPLTP<br>NDKIDRKALPEPDLTANQSQAAYHPPRTETESILVSIWQNVLGIEKIGIRD<br>NFYSLGGDSIQAIQVVARLHSYQLKLETKDLLNYPTIEQVALFVKSTTR<br>KSDQGIIAGNVPLTPIQKWFFGKNFTNTGHWNQSSVLYRPEGFDPKVIQ<br>SVMDKIIEHHDALRMVYQHENGNVVQHNRGLGGQLYDFFSYNLTAQP<br>DVQQAIEAETQRLHSSMNLQEGPLVKVALFQTLHGDHLFLAIHHLVVD<br>GISWRILFEDLATGYAQALAGQAISLPEKTDSFQSWSQWLQEYANEADL<br>LSEIPYWESLESQAKNVSLPKDYEVTDCKQKSVRNMRIRLHPEETEQLL<br>KHANQAYQTEINDLLLAALGLAFAEWSKLAQIVIHLEGHGREDIIEQAN<br>VARTVGWFTSQYPVLLDLKQTAPLSDYIKLTKENMRKIPRKGIGYDILK<br>HVTLPENRGSLSFRVQPEVTFNYLGQFDADMRTELFTRSPYSGGNTLGA<br>DGKNNLSPESEVYTALNITGLIEGGELVLTFSYSSEQYREESIQQLSQSYQ<br>KHLLAIIAHCTEKKEVERTPSDFSVKGLQMEEMDDIFELLANTLRGSRS<br>HHHHHH
[TycB1] C-A-PCP-His$_6$	MSVFSKEQVQDMYALTPMQEGMLFHALLDQEHNSHLVQMSISLQGDL<br>DVGLFTDSLHVLVERYDVFRTLFLYEKLKQPLQVVLKQRPIPIEFYGLSA<br>CDESEKQLRYTQYKRADQERTFHLAKDPLMRVALFQMSQHDYQVIWS<br>FHHILMDGWCFSIIFDDLLAIYLSLQNKTALSLEPVQPYSRFINWLEKQN<br>KQAALNYWSDYLEAYEQKTTLPKKEAAFAKAFQPTQYRFSLNRTLTKQ<br>LGTIASQNQVTLSTVIQTIWGVLLQKYNAAHDVLFGSVVSGRPTDIVGI<br>DKMVGLFINTIPFRVQAKAGQTFSELLQAVHKRTLQSQPYEHVPLYDIQ<br>TQSVLKQELIDHLLVIENYPLVEALQKKALNQQIGFTITAVEMFEPTNYD<br>LTVMVMPKEELAFRFDYNAALFDEQVVQKLAGHLQQIADCVANNSGV<br>ELCQIPLLTEAETSQLLAKRTETAADYPAATMHELFSRQAEKTPEQVAV<br>VFADQHLTYRELDEKSNQLARFLRKKGIGTGSLVGTLLDRSLDMIVGIL<br>GVLKAGGAFVPIDPELPAERIAYMLTHSRVPLVVTQNHLRAKVTTPTET<br>IDINTAVIGEESRAPIESLNQPHDLFYIIYTSGTTGQPKGVMLEHRNMAN<br>LMHFTFDQTNIAFHEKVLQYTTCSFDVCYQEIFSTLLSGGQLYLITNELR<br>RHVEKLFAFIQEKQISILSLPVSFLKFIFNEQDYAQSFPRCVKHIITAGEQL<br>VVTHELQKYLRQHRVFLHNHYGPSETHVVTTCTMDPGQAIPELPPIGKP<br>ISNTGIYILDEGLQLKPEGIVGELYISGANVGRGYLHQPELTAEKFLDNP<br>YQPGERMYRTGDLARWLPDGQLEFLGRIDHQVKIRGHRIELGEIESRLL<br>NHPAIKEAVVIDRADETGGKFLCAYVVLQKALSDEEMRAYLAQALPEY<br>MIPSFFVTLERIPVTPNGKTDRRALPKPEGSAKTKADYVAPTTELEQKLV

	AIWEQILGVSPIGIQDHFFTLGGHSLKAIQLISRIQKECQADVPLRVLFEQ PTIQALAAYVEGGGESAYLAIPQAEPQAYYPVSSAQKRMLILNQLDPHS TVYNLPVAMILEGSRSHHHHHH
[TycA-TycB1] SBP-A-PCP-E--C-A-PCP-His_6	MDEKTTGWRGGHVVEGLAGELEQLRARLEHHPQGQREPMVANQANLI DNKRELEQHALVPYAQGKSIHQLFEEQAEAFPDRVAIVFENRRLSYQEL NRKANQLARALLEKGVQTDSIVGVMMEKSIENVIAILAVLKAGGAYVPI DIEYPRDRIQYILQDSQTKIVLTQKSVSQLVHDVGYSGEVVVLDEEQLD ARETANLHQPSKPTDLAYVIYTSGTTGKPKGTMLEHKGIANLQSFFQNS FGVTEQDRIGLFASMSFDASVWEMFMALLSGASLYILSKQTIHDFAAFE HYLSENELTIITLPPTYLTHLTPERITSLRIMITAGSASSAPLVNKWKDKL RYINAYGPTETSICATIWEAPSNQLSVQSVPIGKPIQNTHIYIVNEDLQLL PTGSEGELCIGGVGLARGYWNRPDLTAEKFVDNPFVPGEKMYRTGDLA KWLTDGTIEFLGRIDHQVKIRGHRIELGEIESVLLAHEHITEAVVIAREDQ HAGQYLCAYYISQQEATPAQLRDYAAQKLPAYMLPSYFVKLDKMPLTP NDKIDRKALPEPDLTANQSQAAYHPPRTETESILVSIWQNVLGIEKIGIRD NFYSLGGDSIQAIQVVARLHSYQLKLETKDLLNYPTIEQVALFVKSTTR KSDQGIIAGNVPLTPIQKWFFGKNFTNTGHWNQSSVLYRPEGFDPKVIQ SVMDKIIEHHDALRMVYQHENGNVVQHNRGLGGQLYDFFSYNLTAQP DVQQAIEAETQRLHSSMNLQEGPLVKVALFQTLHGDHLFLAIHHLVVD GISWRILFEDLATGYAQALAGQAISLPEKTDSFQSWSQWLQEYANEADL LSEIPYWESLESQAKNVSLPKDYEVTDCKQKSVRNMRIRLHPEETEQLL KHANQAYQTEINDLLLAALGLAFAEWSKLAQIVIHLEGHGREDIIEQAN VARTVGWFTSQYPVLLDLKQTAPLSDYIKLTKENMRKIPRKGIGYDILK HVTLPENRGSLSFRVQPEVTFNYLGQFDADMRTELFTRSPYSGGNTLGA DGKNNLSPESEVYTALNITGLIEGGELVLTFSYSSEQYREESIQQLSQSYQ KHLLAIIAHCTEKKEVERTPSDFSVKGLQMEEMDDIFELLANTLRGGSV FSKEQVQDMYALTPMQEGMLFHALLDQEHNSHLVQMSISLQGDLDVG LFTDSLHVLVERYDVFRTLFLYEKLKQPLQVVLKQRPIPIEFYDLSACDE SEKQLRYTQYKRADQERTFHLAKDPLMRVALFQMSQHDYQVIWSFHHI LMDGWCFSIIFDDLLAIYLSLQNKTALSLEPVQPYSRFINWLEKQNKQA ALNYWSDYLEAYEQKTTLPKKEAAFAKAFQPTQYRFSLNRTLTKQLGT IASQNQVTLSTVIQTIWGVLLQKYNAAHDVLFGSIVSGRPTDIVGIDKM VGLFINTIPFRVQAKAGQTFSELLQAVHKRTLQSQPYEHVPLYDIQTQSV LKQELIDHLLVIENYPLVEALQKKALNQQIGFTITAVEMFEPTNYDLTV MVMPKEELAFRFDYNAALFDEQVVQKLAGHLQQIADCVANNSGVELC QIPLLTEAETSQLLAKRTETAADYPAATMHELFSRQAEKTPEQVAVVFA DQHLTYRELDEKSNQLARFLRKKGIGTGSLVGTLLDRSLDMIVGILGVL KAGGAFVPIDPELPAERIAYMLTHSRVPLVVTQNHLRAKVTTPTETIDIN TAVIGEESRAPIESLNQPHDLFYIIYTSGTTGQPKGVMLEHRNMANLMH FTFDQTNIAFHEKVLQYTTCSFDVCYQEIFSTLLSGGQLYLITNELRRHV EKLFAFIQEKQISILSLPVSFLKFIFNEQDYAQSFPRCVKHIITAGEQLVVT HELQKYLRQHRVFLHNHYGPSETHVVTTCTMDPGQAIPELPPIGKPISNT GIYILDEGLQLKPEGIVGELYISGANVGRGYLHQPELTAEKFLDNPYQPG ERMYRTGDLARWLPDGQLEFLGRIDHQVKIRGHRIELGEIESRLLNHPAI KEAVVIDRADETGGKFLCAYVVLQKALSDEEMRAYLAQALPEYMIPSF FVTLERIPVTPNGKTDRRALPKPEGSAKTKADYVAPTTELEQKLVAIWE QILGVSPIGIQDHFFTLGGHSLKAIQLISRIQKECQADVPLRVLFEQPTIQA LAAYVEGSRSHHHHHH

SUPPLEMENTARY FIGURES

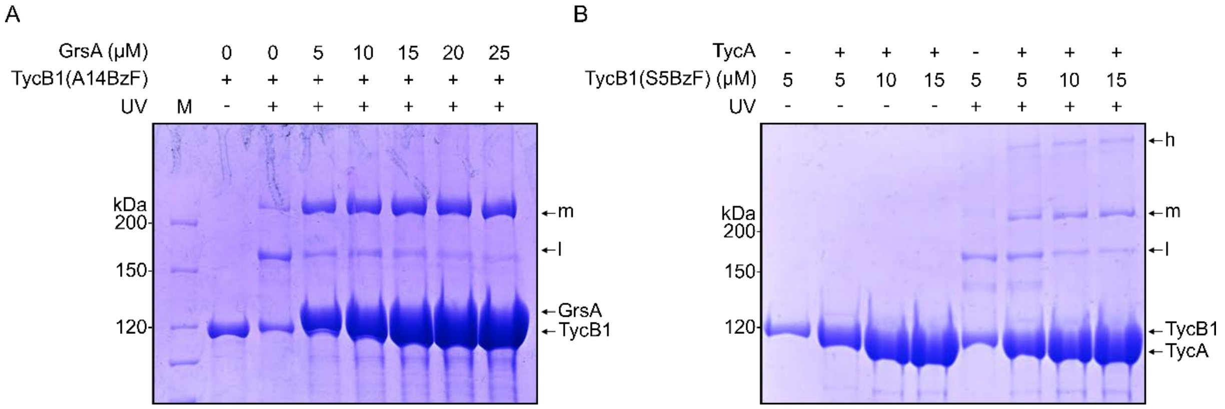

Figure S1. Effect of protein concentration on pattern of photo-crosslinking. Shown are coomassie-stained SDS-PAGE gels. A) 5 µM of TycB1(A14BzF) were incubated at 25 °C for 45 min with varying concentrations of GrsA (0 to 25 µM) and irradiated with 366 nm for 45 min. B) 5 µM of TycA were incubated at 25 °C for 45 min with varying concentrations of TycB1(S5BzF) (0 to 15 µM) and irradiated with 366 nm for 45 min. h = high band, m = middle band, l = low band. Calculated masses are 132.5 kDa (GrsA), 125.2 kDa (TycB1) and 123.9 kDa (TycA).

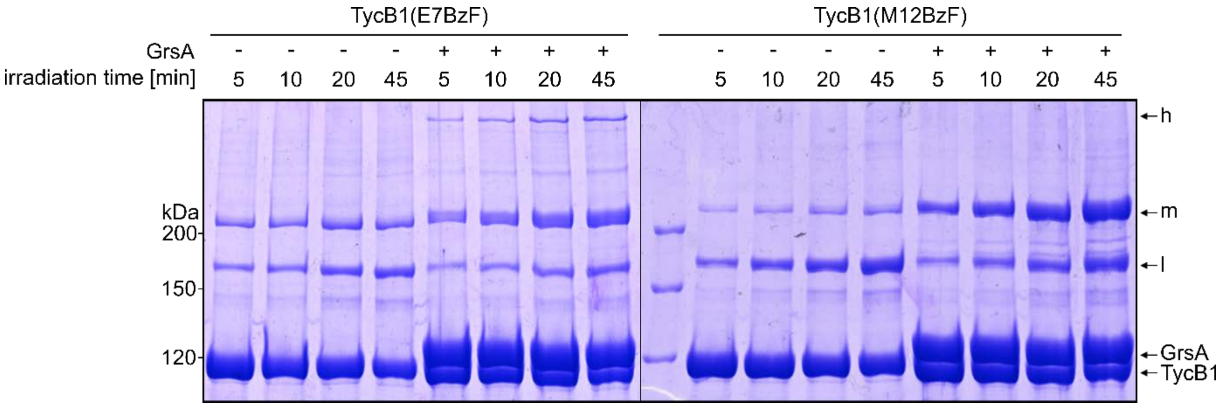

Figure S2. Effect of irradiation time. Shown is a coomassie-stained SDS-PAGE gel. TycB1(E7BzF) and TycB1(M12BzF) in presence of partner protein GrsA (each at 5 µM) were incubated at 25 °C for 45 min and irradiated at 366 nm for different periods of time. h = high band, m = middle band, l = low band. Calculated masses are 132.5 kDa (GrsA) and 125.2 kDa (TycB1).

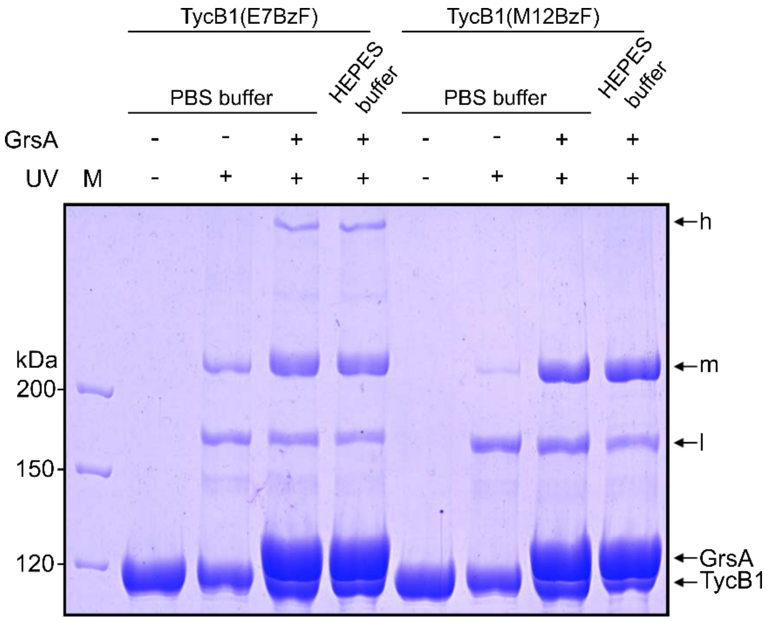

Figure S3. Variation of buffer in photo-crosslink assays. Shown is a coomassie-stained SDS-PAGE gel. Photo-crosslink reactions between TycB1(E7BzF) and TycB1(M12BzF), respectively, with partner protein GrsA (each at 5 µM) were performed in PBS buffer (140 mM NaCl, 2.7 mM KCl, 1.5 mM KH_2PO_4, 8.1 mM Na_2HPO_4, pH 7.4). The results were virtually indistinguishable from those obtained when the experiments were regularly performed in 50 mM HEPES, 100 mM NaCl, 10 mM $MgCl_2$, 1 mM EDTA, pH 7.0, samples of which are loaded in control lanes for comparison. These findings ruled out that the C-H bonds of HEPES at the high buffer concentrations participate in the photo-crosslinking reaction and partially quench the protein-protein crosslinking. h = high band, m = middle band, l = low band. Calculated masses are 132.5 kDa (GrsA) and 125.2 kDa (TycB1).

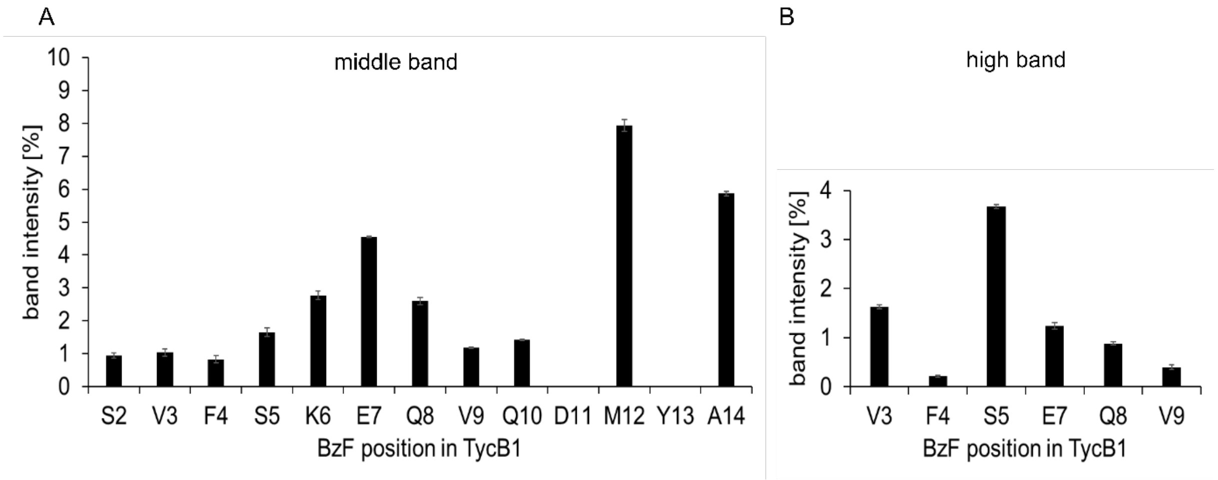

Figure S4. Densitometric analysis of SDS-PAGE crosslink band intensities. The intensities of the indicated bands were calculated as percentage of the entire protein band intensities in the same sample (i.e., sum of TycB1, GrsA, l, m, and h bands). (A) Crosslink band at approx. 240 kDa. The background intensity of the m band in the respective GrsA negative control was subtracted for this analysis. (B) Crosslink band with a migration behavior at high molecular weight.

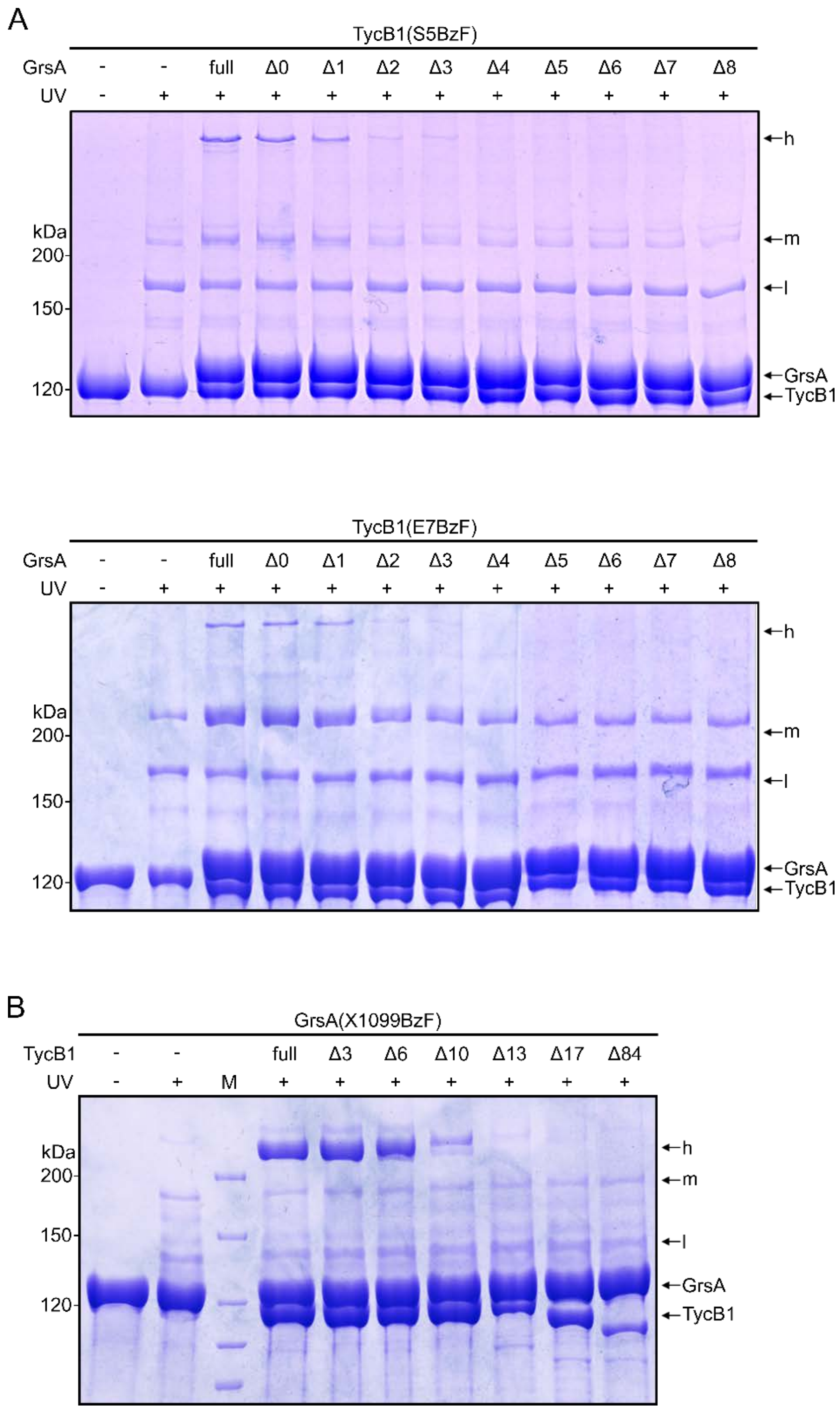

Figure S5. Effect of COM domain truncations on photo-crosslinking activity. Shown are coomassie-stained SDS-PAGE gels. A) Analysis of photo-crosslinking reactions between TycB1(S5BzF) (top panel) and TycB1(E7BzF) (bottom panel) with full-length GrsA (including a C-terminal His-tag) and a series of gradual C-terminal deletion constructs of GrsA (lacking the C-terminal His-tag and an increasing number of C-terminal amino acids from the wildtype sequence).[6] Each protein was at 5 µM concentration and irradiation at 366 nm was performed for 45 min at 25 °C. B) Analysis of the photo-crosslinking reaction between GrsA(X1099BzF) (see Ref[6]) and full-length TycB1 along with a series of N-terminal truncation mutants of TycB1. h = high band, m = middle band, l = low band.

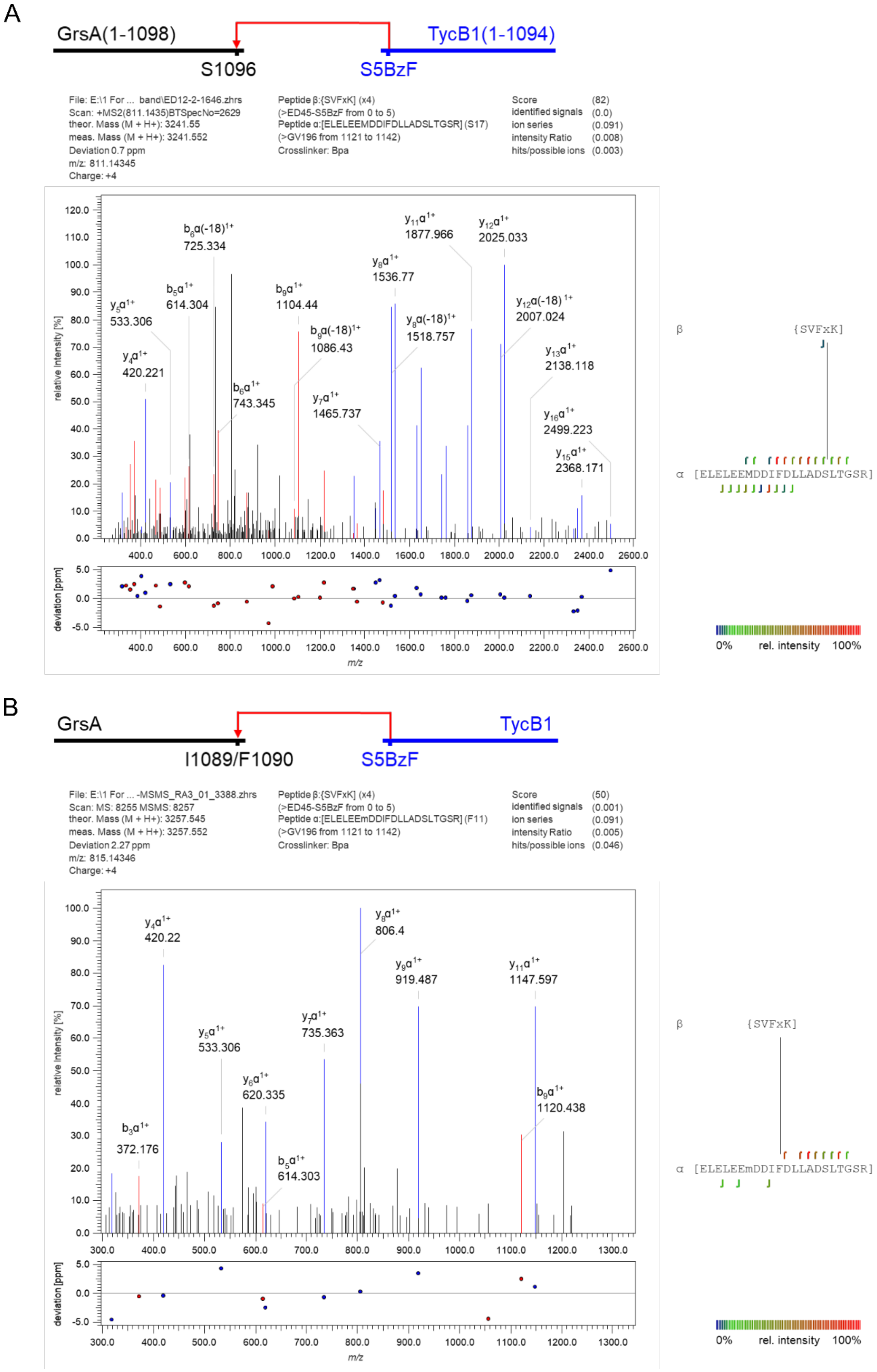
A
GrsA(1-1098)
TycB1(1-1094)
S1096
S5BzF
File: E:\1 For ... band\ED12-2-1646.zhrs
Scan: +MS2(811.1435)BTSpecNo=2629
theor. Mass (M + H+): 3241.55
meas. Mass (M + H+): 3241.552
Deviation 0.7 ppm
m/z: 811.14345
Charge: +4
Peptide β:{SVFxK] (x4)
(>ED45-S5BzF from 0 to 5)
Peptide α:[ELELEEMDDIFDLLADSLTGSR] (S17)
(>GV196 from 1121 to 1142)
Crosslinker: Bpa
Score (82)
identified signals (0.0)
ion series (0.091)
intensity Ratio (0.008)
hits/possible ions (0.003)
relative intensity [%]
deviation [ppm]
m/z
β {SVFxK]
α [ELELEEMDDIFDLLADSLTGSR]
0% rel. intensity 100%
B
GrsA
TycB1
I1089/F1090
S5BzF
File: E:\1 For ... -MSMS_RA3_01_3388.zhrs
Scan: MS: 8255 MSMS: 8257
theor. Mass (M + H+): 3257.545
meas. Mass (M + H+): 3257.552
Deviation 2.27 ppm
m/z: 815.14346
Charge: +4
Peptide β:{SVFxK] (x4)
(>ED45-S5BzF from 0 to 5)
Peptide α:[ELELEEmDDIFDLLADSLTGSR] (F11)
(>GV196 from 1121 to 1142)
Crosslinker: Bpa
Score (50)
identified signals (0.001)
ion series (0.091)
intensity Ratio (0.005)
hits/possible ions (0.046)
relative intensity [%]
deviation [ppm]
m/z
β {SVFxK]
α [ELELEEmDDIFDLLADSLTGSR]
0% rel. intensity 100%

C

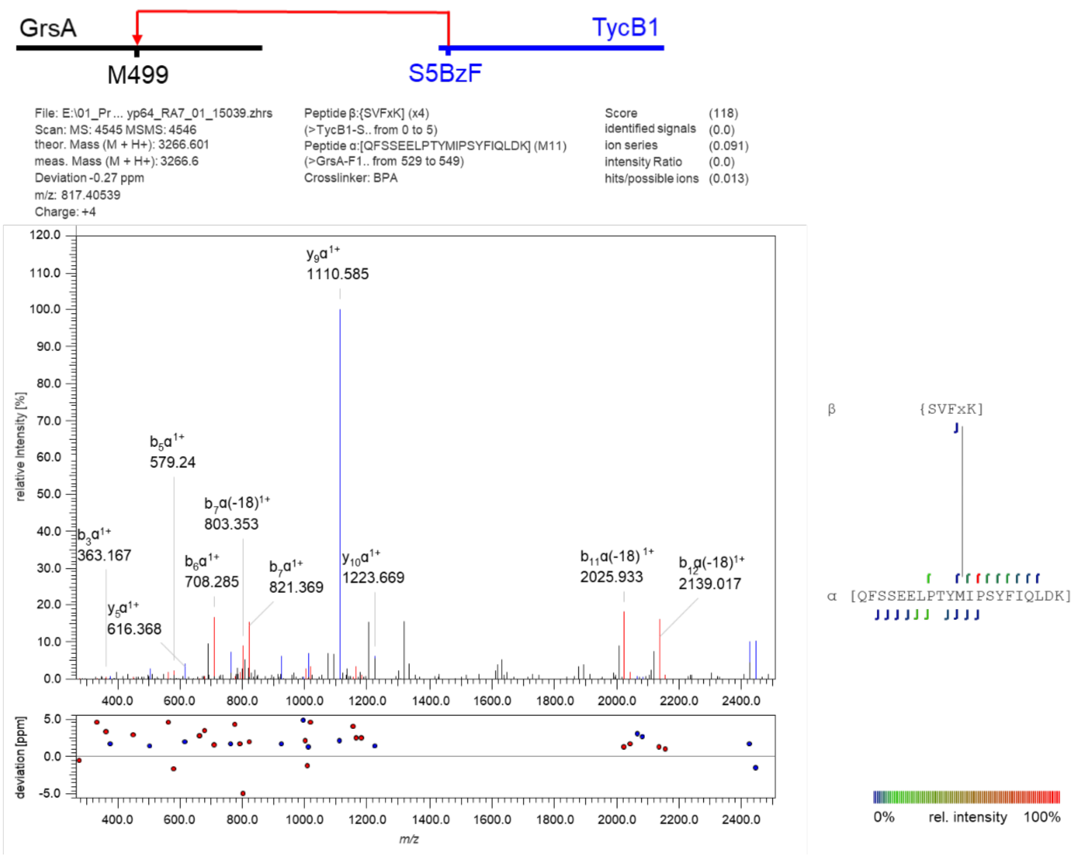

Figure S6. MS/MS mapping analysis of photo-crosslinked peptides. The shown MS/MS spectra are consistent with the expected fragmentation patterns of the illustrated crosslinked peptides, identified using StavroX 3.6.6.[7] (A) Assignment of a crosslink between GrsA (α) and TycB1(S5BzF) (β) peptides from the middle band of the photo reaction. x denotes BzF. The precursor ion $[M+4H]^{4+}$ at m/z 811.1434 matches the expected mass of the crosslinked peptide with a deviation of 0.7 ppm. The GrsA fragment encompasses amino acids E^{1080}LELEEMDDIFDLLADSLT^{1098} and the additional residues GSR from the fused tag. (B) Assignment of a crosslink between GrsA (α) and TycB1(S5BzF) (β) peptides from the middle band of the photo reaction. x denotes BzF. The precursor ion $[M+4H]^{4+}$ at m/z 815.1434 matches the expected mass of the crosslinked peptide with a deviation of 2.3 ppm. The GrsA fragment encompasses amino acids E^{1080}LELEEMDDIFDLLADSLT^{1098} and the additional residues GSR from the fused tag. (C) Assignment of a crosslink between GrsA (α) and TycB1(S5BzF) (β) peptides from the high band of the photo reaction. x denotes BzF. The precursor ion $[M+4H]^{4+}$ at m/z 817.4054 matches the expected mass of the cross-linked peptide with a deviation of -0.3 ppm. The GrsA fragment encompasses amino acids Q^{489}FSSEELPTYMIPSYFIQLDK^{509}.

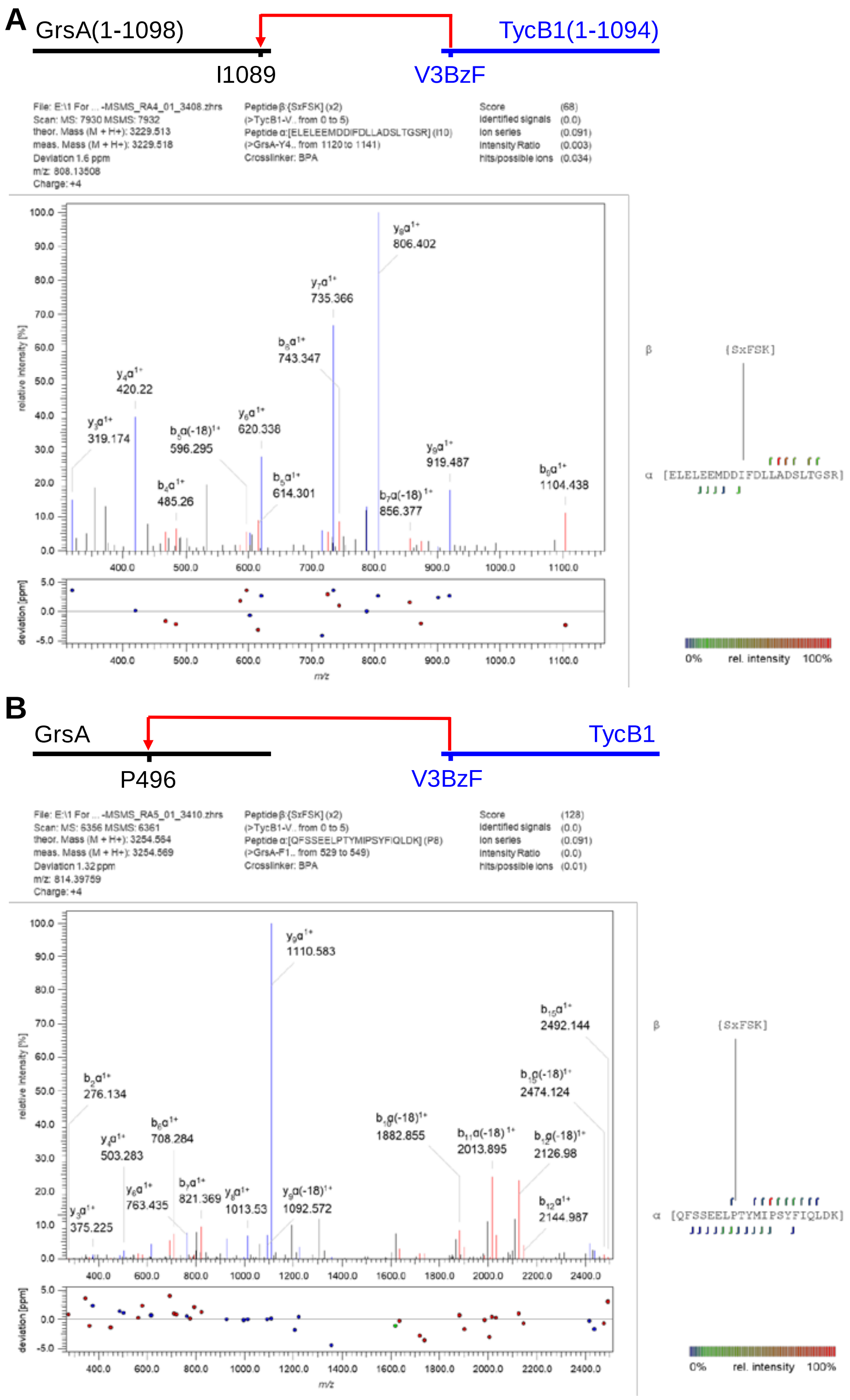

Figure S7. MS/MS spectra of crosslink peptides. (A) Assignment of a crosslink between GrsA (α) and TycB1(V3BzF) (β) peptides from the middle band of the photo reaction. The precursor ion $[M+4H]^{4+}$ at m/z 808.1351 matches the expected mass of the crosslinked peptide to 1.6 ppm. The shown MS/MS spectrum is consistent with the expected fragmentation pattern. (B) Assignment of a crosslink between GrsA (α) and TycB1(V3BzF) (β) peptides from the high band of the photo reaction. X denote BzF, respectively. The precursor ion $[M+4H]^{4+}$ at m/z 814.3976 matches the expected mass of the crosslinked peptide to 1.3 ppm. The shown MS/MS spectrum is consistent with the expected fragmentation pattern.

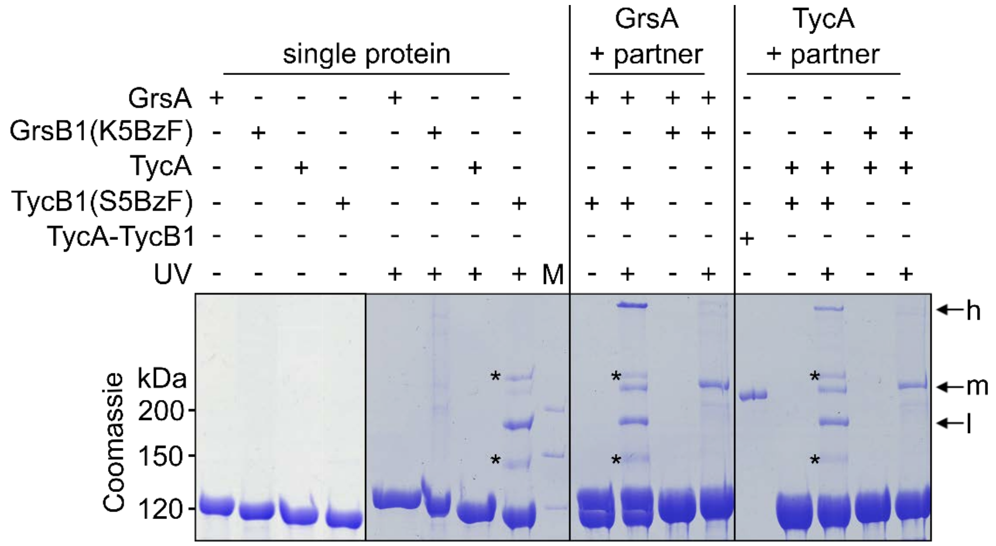

Figure S8. Crosslink experiment with GrsB1 mutant K5BzF and TycB1 mutant S5BzF in the absence and presence of the native partner GrsA and TycA. Shown are coomassie-stained SDS-PAGE gels. The crosslink reactions were incubated for 45 min at 25 °C, followed by irradiation with UV light (λ=366 nm) for 45 min. TycA-TycB1 fusion construct is used as an indicator for the migration behavior of the L-form isomer. Bands marked with an asterisk represent two additional BzF and irradiation dependent crosslink products that were produced by some of the TycB1(BzF) mutants also in the absence of GrsA. In contrast to the other crosslink products, the intensity of these bands varied depending on the protein preparation, suggesting the crosslinks are caused by partially misfolded protein populations. In Figure 3A of the main text the same bands can be seen with weaker intensity. h = high band, m = middle band, l = low band. Calculated masses are 132.5 kDa (GrsA), 125.2 kDa (TycB1), 123.9 kDa (TycA), 122.9 kDa (GrsB1) and 246.7 kDa (TycA-TycB1).

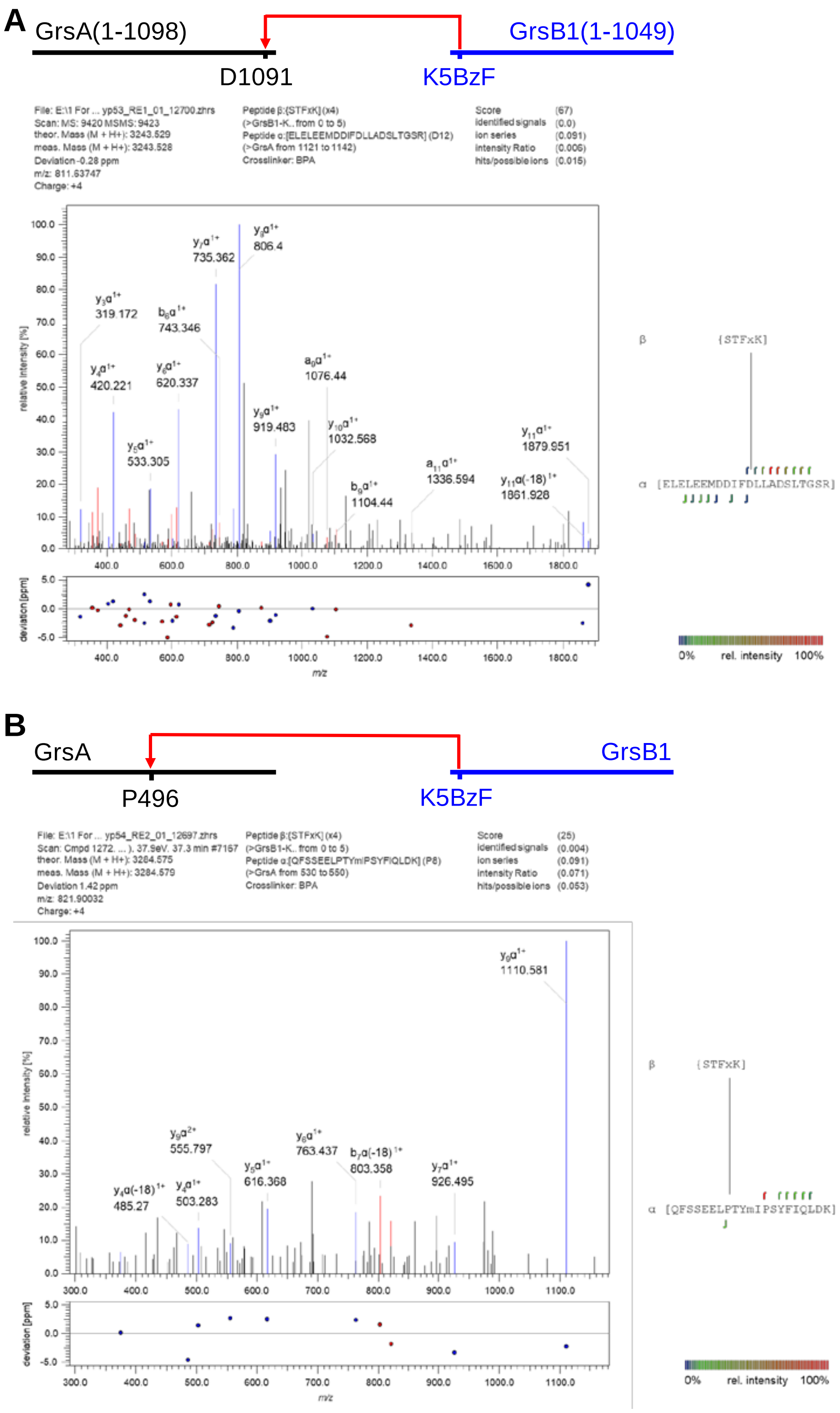

Figure S9. MS/MS spectra of crosslink peptides. (A) Assignment of a crosslink between GrsA (α) and GrsB1(K5BzF) (β) peptides from the middle band of the photo reaction. The precursor ion $[M+4H]^{4+}$ at m/z 811.6375 matches the expected mass of the crosslinked peptide to -0.3 ppm. The shown MS/MS spectrum is consistent with the expected fragmentation pattern. (B) Assignment of a crosslink between GrsA (α) and GrsB1(K5BzF) (β) peptides from the high band of the photo reaction. X denote BzF, respectively. The precursor ion $[M+4H]^{4+}$ at m/z 821.9003 matches the expected mass of the crosslinked peptide to 1.4 ppm. The shown MS/MS spectrum is consistent with the expected fragmentation pattern.

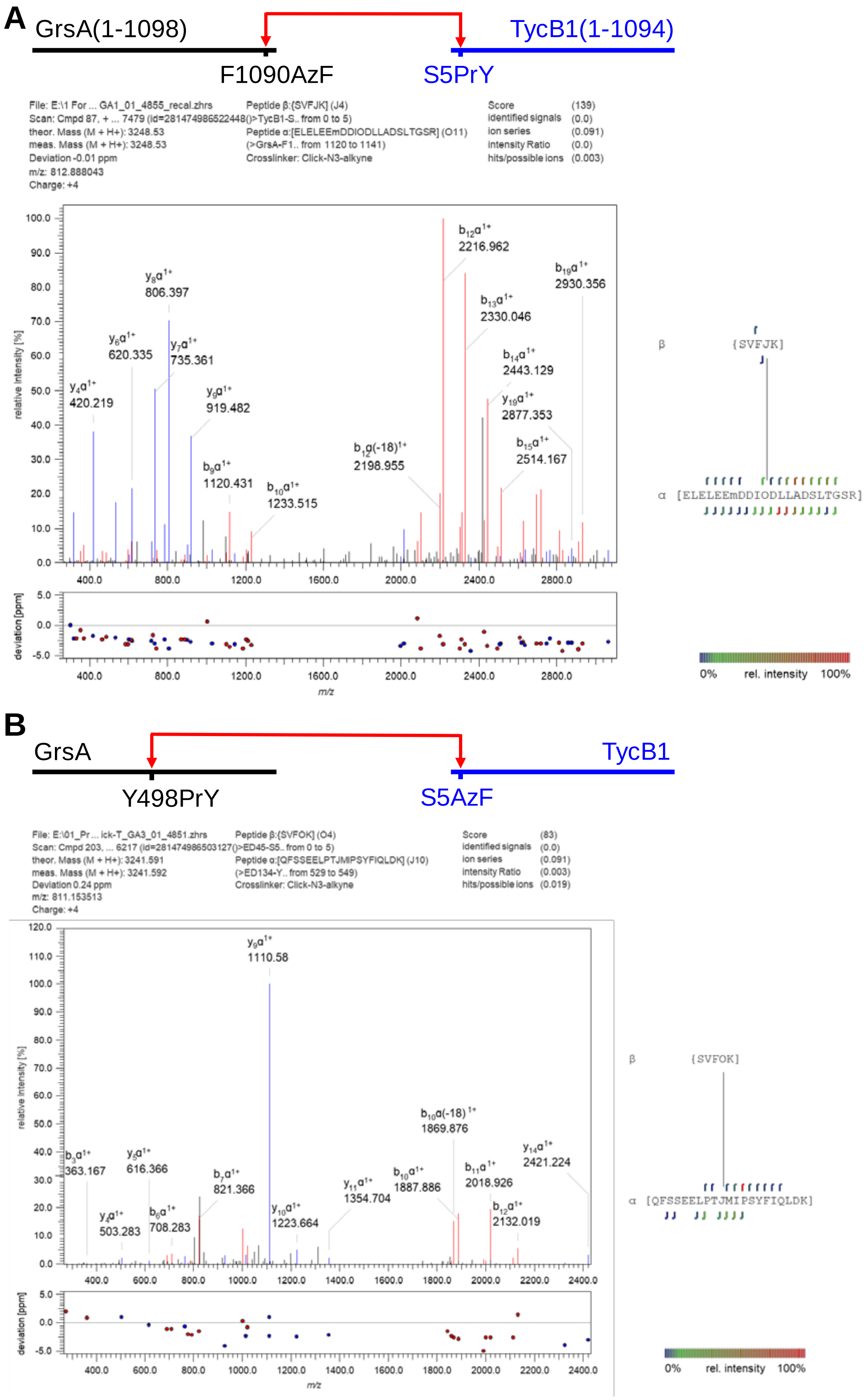
A
GrsA(1-1098)
TycB1(1-1094)
F1090AzF
S5PrY
File: E:\1 For ... GA1_01_4855_recal.zhrs
Scan: Cmpd 87, + ... 7479 (id=281474986522448()>TycB1-S.. from 0 to 5)
theor. Mass (M + H+): 3248.53
meas. Mass (M + H+): 3248.53
Deviation -0.01 ppm
m/z: 812.888043
Charge: +4
Peptide β:{SVFJK} (J4)
Peptide α:[ELELEEmDDIODLLADSLTGSR] (O11)
(>GrsA-F1.. from 1120 to 1141)
Crosslinker: Click-N3-alkyne
Score (139)
identified signals (0.0)
ion series (0.091)
intensity Ratio (0.0)
hits/possible ions (0.003)
relative intensity [%]
deviation [ppm]
m/z
β {SVFJK}
α [ELELEEmDDIODLLADSLTGSR]
0% rel. intensity 100%
B
GrsA
TycB1
Y498PrY
S5AzF
File: E:\01_Pr ... ick-T_GA3_01_4851.zhrs
Scan: Cmpd 203, ... 6217 (id=281474986503127()>ED45-S5.. from 0 to 5)
theor. Mass (M + H+): 3241.591
meas. Mass (M + H+): 3241.592
Deviation 0.24 ppm
m/z: 811.153513
Charge: +4
Peptide β:{SVFOK} (O4)
Peptide α:[QFSSEELPTJMIPSYFIQLDK] (J10)
(>ED134-Y.. from 529 to 549)
Crosslinker: Click-N3-alkyne
Score (83)
identified signals (0.0)
ion series (0.091)
intensity Ratio (0.003)
hits/possible ions (0.019)
relative intensity [%]
deviation [ppm]
m/z
β {SVFOK}
α [QFSSEELPTJMIPSYFIQLDK]
0% rel. intensity 100%

C

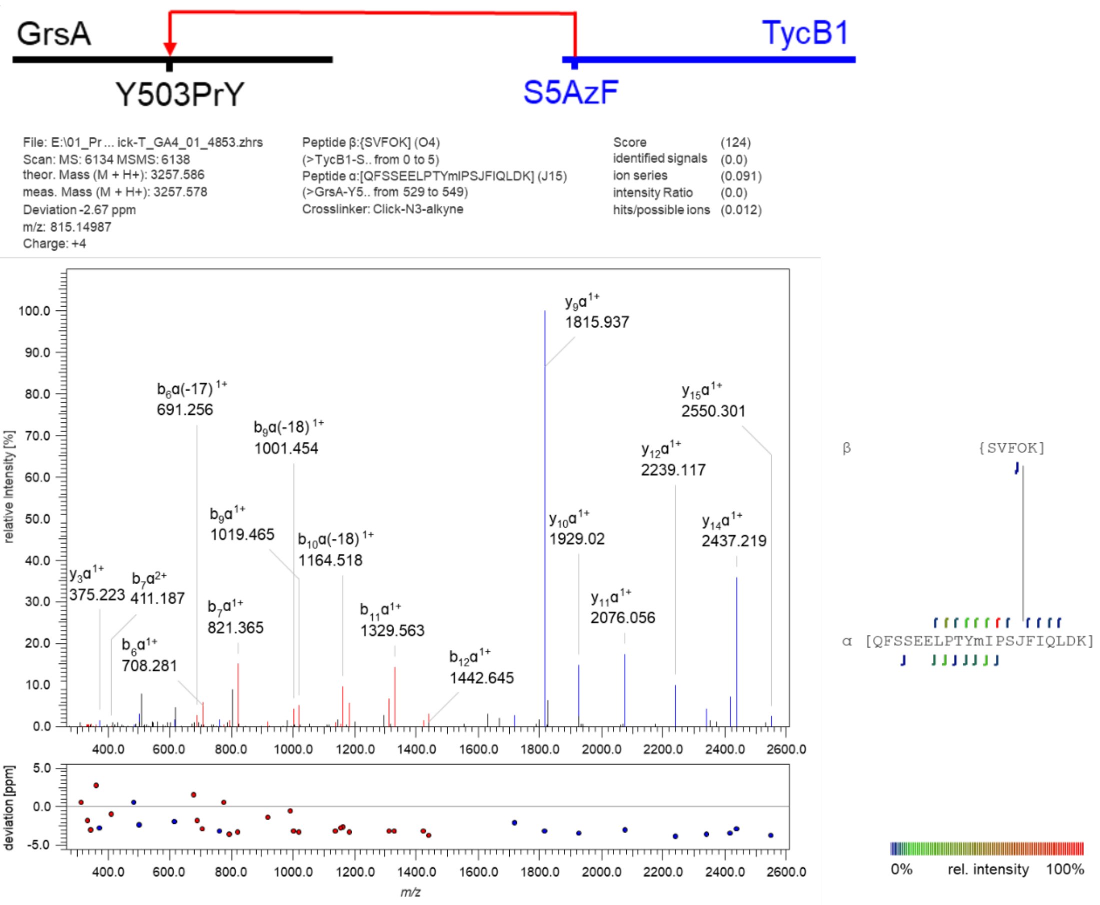

Figure S10. MS/MS spectra of click conjugation products. (A) Assignment of a cross-link between GrsA(F1090AzF) (α) and TycB1(S5PrY) (β) peptides from the middle band of the CuAAC reaction. O and J denote AzF and PrY, respectively. The precursor ion $[M+4H]^{4+}$ at m/z 812.8880 matches the expected mass of the cross-linked peptide with a deviation of 0.0 ppm, and the shown MS/MS spectrum is consistent with the expected fragmentation pattern. (B) Assignment of a cross-link between GrsA(Y498PrY) (α) and TycB1(S5AzF) (β) peptides from the high band of the CuAAC reaction. O and J denote AzF and PrY, respectively. The precursor ion $[M+4H]^{4+}$ at m/z 811.1535 matches the expected mass of the cross-linked peptide with a deviation of 0.2 ppm, and the shown MS/MS spectrum is consistent with the expected fragmentation pattern. (C) Assignment of a crosslink between GrsA(Y503PrY) (α) and TycB1(S5AzF) (β) peptides from the high band of the CuAAC reaction. O and J denote AzF and PrY, respectively. The precursor ion $[M+4H]^{4+}$ at m/z 815.1499 matches the expected mass of the cross-linked peptide with a deviation of -2.7 ppm, and the shown MS/MS spectrum is consistent with the expected fragmentation pattern.

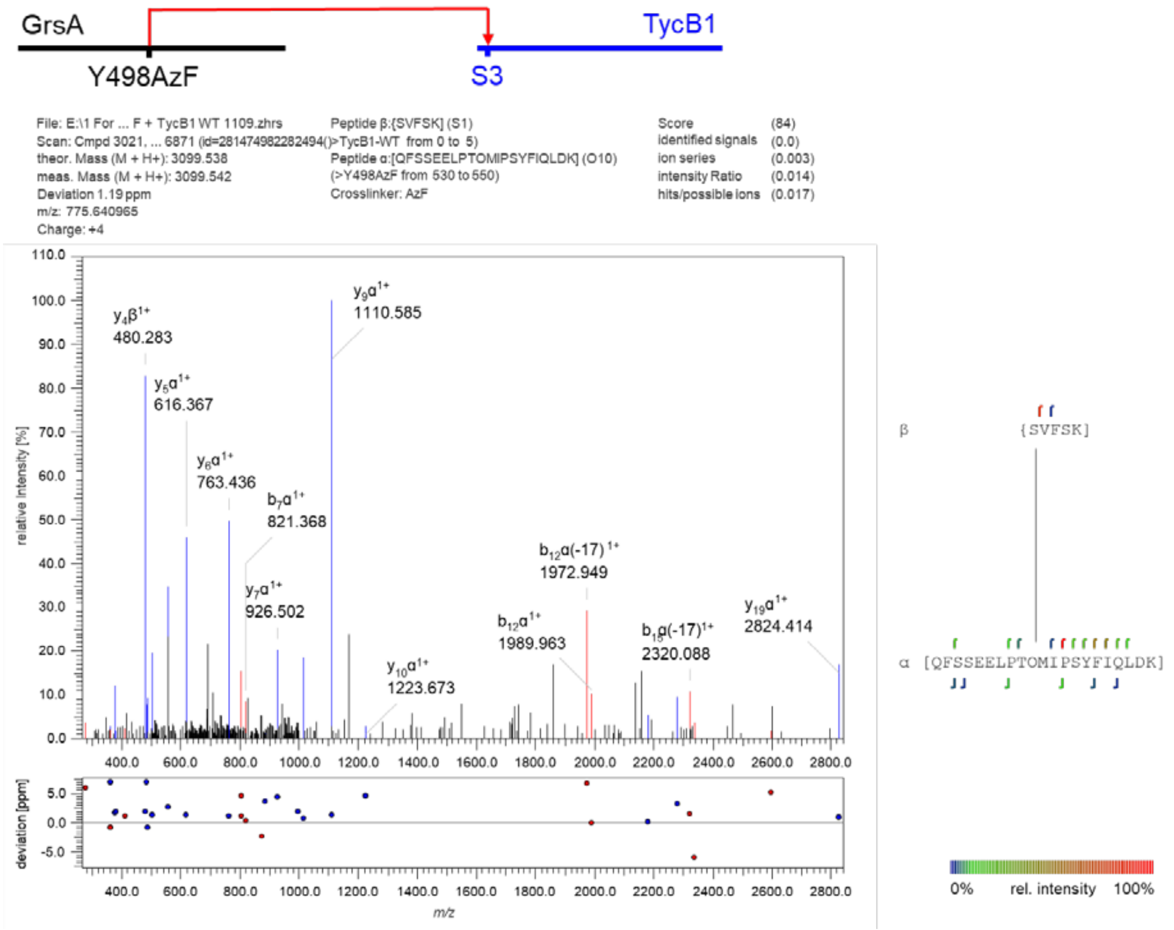

Figure S11. MS/MS mapping analysis of photo-crosslinked peptides. Assignment of a cross-link between GrsA(Y498AzF) (α) and TycB1 (β) peptides from the high band of the photo reaction. O denotes AzF. The precursor ion $[M+4H]^{4+}$ at m/z 779.6391 matches the expected mass of the cross-linked peptide with a deviation of 0.4 ppm. The TycB1 fragment encompasses amino acids S^2VFSK^6.

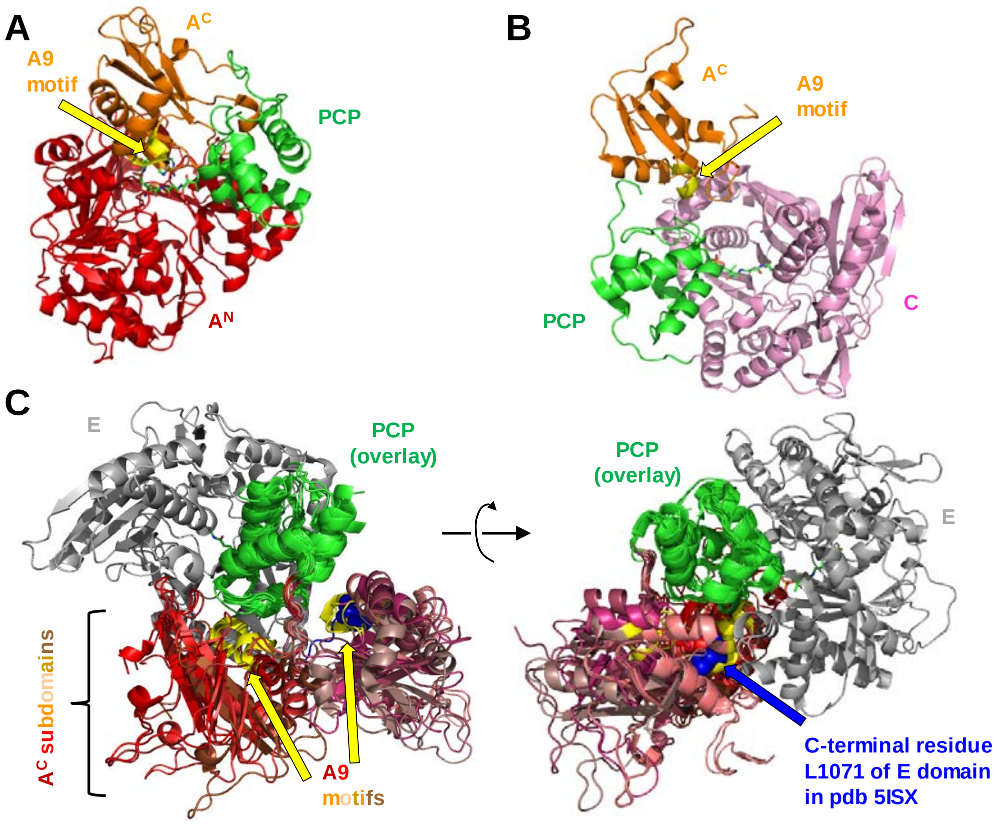

Figure S12. Structural modeling to rationalize proximity between A9 motif and acceptor COM domain. (A) Crystal structure of an A^N-A^C-PCP unit in transfer conformation (pdb: 4ZXJ). The A9 motif corresponding to the GrsA sequence PTYMI is colored in yellow. The A^N domain is shown in red, the A^C subdomain in orange, and the PCP in green. The Ppant arm is shown in stick representation. (B) Crystal structure of an A^C-PCP-C unit in donor condensation conformation (pdb: 6MFX). The A9 motif corresponding to the GrsA sequence PTYMI is colored in yellow. The A^C subdomain is shown in orange and the C domain is shown in light pink. The Ppant arm is shown in stick representation. (C) Structural model (partial) of the unknown epimerization conformation. The PCP-E structure (pdb: 5ISX) was structurally aligned on the PCP with the A^C-PCPs units from all known NRPS structures in which the PCP binds to a catalytic domain other than an A domain (see Tab. S1) to visualize all currently known orientations of the A^C subdomain relative to the PCP. Since the PCP-E structure lacks electron density for the C-terminal residues 1071-1098 of GrsA, the location of the donor COM domain (~ aa 1073-1098) and thus the binding site of the acceptor COM domain can only be extrapolated from the last reference point L1071, which is depicted as blue spheres. Further uncertainty is added by the unknown orientation of the acceptor COM domain with the photo-crosslinking moiety. Nevertheless, this structural and modeling analysis suggests that a proximity between the A9 motif of the A^C subdomain and the COM binding interface is plausible (see close proximity of blue spheres with yellow A9 motif in one subset of possible A^C localizations). Together, we assume our mapped crosslink from the acceptor COM domain into the A9 motif is consistent with the available structural data and most likely reports on the epimerization conformation in the GrsA/TycB1 complex. The E domain is shown in grey, the PCP in green and the A^C subdomains in reddish colors. The A9 motif corresponding to the GrsA sequence PTYMI is colored in yellow for each A^C subdomain structure. All images were created with PyMol.

ELECTRONIC SUPPLEMENTARY REFERENCES

1. E. J. Drake, B. R. Miller, C. Shi, J. T. Tarrasch, J. A. Sundlov, C. L. Allen, G. Skiniotis, C. C. Aldrich and A. M. Gulick, *Nature*, 2016, **529**, 235-238.
2. J. M. Reimer, M. N. Aloise, P. M. Harrison and T. M. Schmeing, *Nature*, 2016, **529**, 239-242.
3. A. Tanovic, S. A. Samel, L. O. Essen and M. A. Marahiel, *Science*, 2008, **321**, 659-663.
4. D. F. Kreitler, E. M. Gemmell, J. E. Schaffer, T. A. Wencewicz and A. M. Gulick, *Nat Commun*, 2019, **10**, 3432.
5. J. M. Reimer, M. Eivaskhani, I. Harb, A. Guarne, M. Weigt and T. M. Schmeing, *Science*, 2019, **366**.
6. E. Dehling, G. Volkmann, J. C. Matern, W. Dorner, J. Alfermann, J. Diecker and H. D. Mootz, *J Mol Biol*, 2016, **428**, 4345-4360.
7. M. Gotze, J. Pettelkau, S. Schaks, K. Bosse, C. H. Ihling, F. Krauth, R. Fritzsche, U. Kuhn and A. Sinz, *J Am Soc Mass Spectrom*, 2012, **23**, 76-87.
